# Photo-induced ultrafast active ion transport through graphene oxide membranes

**DOI:** 10.1038/s41467-019-09178-x

**Published:** 2019-03-12

**Authors:** Jinlei Yang, Xiaoyu Hu, Xian Kong, Pan Jia, Danyan Ji, Di Quan, Lili Wang, Qi Wen, Diannan Lu, Jianzhong Wu, Lei Jiang, Wei Guo

**Affiliations:** 1 CAS Key Laboratory of Bio-inspired Materials and Interfacial Science, Technical Institute of Physics and Chemistry, Chinese Academy of Sciences, Beijing, 100190 PR China; 2 University of Chinese Academy of Sciences, Beijing, 100049 PR China; 3 State Key Joint Laboratory of Chemical Engineering, Department of Chemical Engineering, Tsinghua University, Beijing, 100084 PR China; 4 Department of Chemical and Environmental Engineering, University of California, Riverside, CA 92521 USA

## Abstract

Layered graphene oxide membranes (GOM) with densely packed sub-nanometer-wide lamellar channels show exceptional ionic and molecular transport properties. Mass and charge transport in existing materials follows their concentration gradient, whereas attaining anti-gradient transport, also called active transport, remains a great challenge. Here, we demonstrate a coupled photon-electron-ion transport phenomenon through the GOM. Upon asymmetric light illumination, cations are able to move thermodynamically uphill over a broad range of concentrations, at rates much faster than that via simple diffusion. We propose, as a plausible mechanism, that light irradiation reduces the local electric potential on the GOM following a carrier diffusion mechanism. When the illumination is applied to an off-center position, an electric potential difference is built that can drive the transport of ionic species. We further develop photonic ion switches, photonic ion diodes, and photonic ion transistors as the fundamental elements for active ion sieving and artificial photosynthesis on synthetic nanofluidic circuits.

## Abstract (web summary)

Ionic transport through subnanometer-sized channels in 2D material-based membranes can be exploited for energy and separation applications. Here the authors demonstrate the visible light activation of an ultrafast ionic flux against a concentration gradient in graphene oxide membranes.

## Introduction

Liquid processing of colloidal 2D materials provides a facile and scalable way to produce membrane materials with densely packed sub-nanometer-wide channels for sustainable energy, environmental, and healthcare applications^1–8^. Novel transport phenomena occurred in the angstrom range allows fast and precise sieving of ionic and molecular species through the interstitial space between restacked 2D nanosheets^9,10^. However, mass and charge transport in existing membrane materials follows their concentration gradient^11,12^. Attaining anti-gradient transport (viz., active transport), as effective as natural counterparts^13,14^, remains a great challenge in fully abiotic micro- or nanosystems^15,16^.

Existing artificial molecular transport systems reside in lipid or liquid membranes, and use the energy of light to pump protons or metal ions through the membrane against their concentration gradients^17–19^. However, the supported liquid membranes become the bottleneck for practical applications, because they are fragile and hardly compatible with other components^20^. Moreover, these molecular transport systems hinge on much larger ion-binding shuttle molecules for transmembrane ion transport^21^. In this case, a large portion of energy is spent on the movement of the shuttle molecule, which makes the ion transport process less efficient. A shuttle-free ion pumping system in solid-state materials is therefore needed to solve such problems.

Here, we report the generation of a net cationic flow through layered graphene oxide membrane (GOM) upon asymmetric light illumination. Against a concentration gradient, cations are moved thermodynamically uphill at rates orders of magnitude faster than that via simple diffusion. We propose a plausible mechanism to explain this phenomenon based on a carrier diffusion model and molecular dynamics (MD) simulations. Following the mechanism, we further develop photonic ion switches (PIS), photonic ion diodes (PID), and photonic ion transistors (PIT) as the fundamental elements for active ion sieving and artificial photosynthesis on synthetic nanofluidic circuits.

## Results

### Device and transport phenomenon

**Fig. 1:**
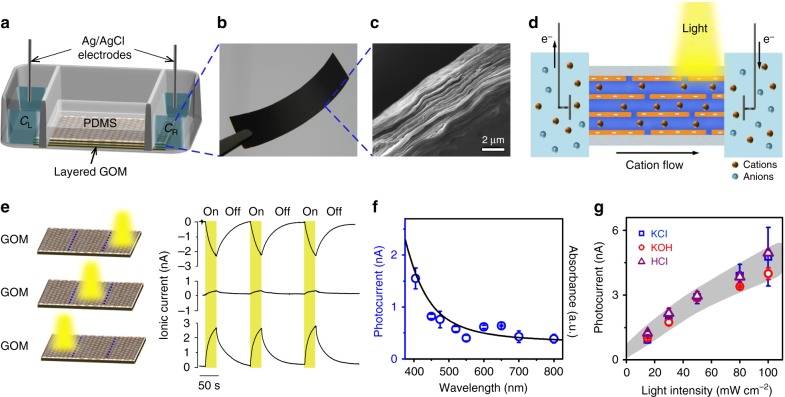
Photo-induced ion transport through GOM. **a** Scheme of the device. **b** A photograph of the GO strip. **c** SEM image on the cross-section shows a layered structure. **d** Schematic illustration of the generation of net ionic flow through the GOM upon asymmetric light illumination. **e** Time traces of the photocurrent. Light illumination (100 mW cm^−2^, 30 s duration) was applied separately on three different positions (right, middle, or left). **f** UV–vis absorption spectrum of GO dispersion (solid curve), and photocurrent at each wavelength (open circles). **g** Similar photo-response is found in KCl, KOH, or HCl solutions. The electrolyte concentrations were 1 μM. Error bars denote standard deviation

The GOMs were fabricated by vacuum filtration of liquid exfoliated GO nanosheets, followed by a mild thermal annealing process to improve their water stability^22^. The GOM is about 5-μm-thick (Fig. 1a, b). Scanning electron microscopy (SEM) observation shows a densely layered structure (Fig. 1c). The interlayer spacing (*d*) of wet GOMs is about 1.26 nm (Supplementary Note 1 and Supplementary Fig. 1). Taking into account the thickness of GO sheet (~0.34 nm), the effective height of the lamellar nanochannels is about 0.92 nm, allowing for the transport of water and most hydrated ions^9^. A piece of rectangular GO strip (15 mm × 9 mm) was embedded in a transparent polydimethylsiloxane (PDMS) elastomer (Fig. 1a). The two sides of the sealed GOM were trimmed off to open the lateral ends. Two solution reservoirs were built on the two ends of the GOM. 3.5 ml ionic solution with concentrations of *C*_L_ and *C*_R_ was filled in each reservoir. Ag/AgCl electrodes were used to record the horizontal ion transport through the GOM^23^.

With equivalent electrolyte solution (KCl, 1 μΜ) placed in the two reservoirs, we discover a synchronous photo-electric response without externally applied voltage, when the GO strip was irradiated locally by simulated sunlight from a xenon lamp (Fig. 1d). The recorded ionic current flows from the non-illuminated region to the illuminated region. For example, once the illumination (light intensity ~100 mW cm^−2^) was applied to the right 1/3 (in length) of the GO strip, the net ionic current arises from zero to ~−2.27 nA within 30 s (Fig. 1e). The direction of the photocurrent is reversed when the illumination shifts to the left 1/3 (in length) of the GO strip, without much altering its magnitude (~+2.54 nA). Surprisingly, if the light beam locates in the middle of the GO strip, no clear photo-response can be found.

The negatively charged sub-nm-wide lamellar channels in GOM are fully covered by the Debye screening layer^24^. The photocurrent consists of nearly perfect cations (*t*_+_ > 0.97, Supplementary Note 2, Supplementary Fig. 2 and Supplementary Table 1). The photocurrent and the incident photon to current efficiency measured at different light wavelength generally agree with the absorption spectrum of GO dispersion (Fig. 1f, Supplementary Note 3 and Supplementary Fig. 3). Direct heating on part of the GOM does not generate net ion transport, and the photocurrent is insensitive to the temperature from 4 to 40 °C (Supplementary Note 4 and Supplementary Figs. 4–5). Although the light illumination may slightly reduce GO, its influence is limited (Supplementary Note 5, Supplementary Figs. 6–10, and Supplementary Tables 2–3). These evidences suggest that the observed photocurrent does not originate from the thermal effect^25^.

Similar photo-response can be found in either neutral, acidic, or alkali electrolytes (Fig. 1g). The magnitude of photocurrent depends on light intensity. Besides GOM, the photo-induced ion transport phenomenon can be found in other types of 2D layered materials reconstructed from, for example, WSe_2_, MoS_2_, WS_2_, and reduced GO (Supplementary Note 6 and Supplementary Fig. 11). As control experiments, we also check the photo-response in pH test papers and cellulose acetate membranes (Supplementary Note 7 and Supplementary Fig. 12). No detectable photo-response was found.

### Anti-gradient transport

**Fig. 2:**
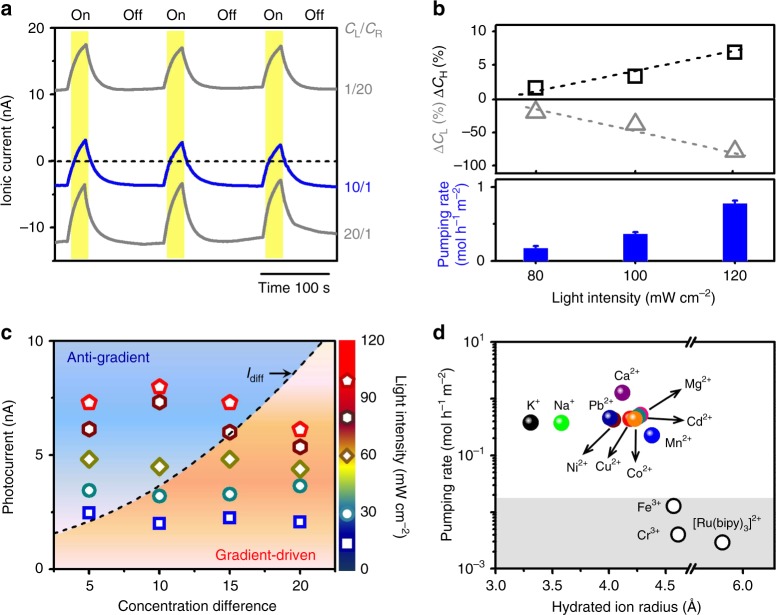
Anti-gradient ion transport. **a** Time traces of photocurrent under concentration gradient. Light illumination (100 mW cm^−2^) was on the left 1/3 of the GOM. When *C*_L_/*C*_R_ = 10/1, the ionic current goes across the zero line, and further increases in the reverse direction, showing anti-gradient ion transport. But when the concentration gradient was too high (e.g., *C*_L_/*C*_R_ = 20/1) or in the reverse direction (*C*_L_/*C*_R_ = 1/20), no ionic current reversion was found. **b** Photo-induced variations in K^+^ concentration and corresponding ion pumping rates measured by ICP-OES. After light illumination for 180 s, *C*_high_ further arises, while *C*_low_ falls down, showing anti-gradient ion transport. Error bars denote standard deviation. **c** Photocurrent against different concentration gradients and with varied light intensities. The dashed line indicates the diffusion current (*I*_diff_). When the magnitude of the photocurrent is larger than *I*_diff_, anti-gradient ion transport can be found. Otherwise, the ion transport is governed by the concentration gradient. **d** Pumping rates for different ionic species against 10-fold concentration gradient. The gray area indicates the below-detection limit of the measurements. The concentration change for Fe^3+^, Cr^3+^, [Ru(bipy)_3_]^2+^ was not effectively detected

More intriguingly, under concentration gradient, anti-gradient ion transport can be realized upon asymmetric light illumination. For example, under a 10-fold concentration gradient (*C*_L_ = 10 μM, *C*_R_ = 1 μM), the initial diffusion current (*I*_diff_) was about −3.67 nA (Fig. 2a). Upon light illumination (100 mW cm^−2^) on the left 1/3 of the GO strip, the ionic current soon goes across the zero line and further increases in the reverse direction, leading to anti-gradient cation transport. By contrast, if the concentration gradient goes in the reverse direction (*C*_L_ < *C*_R_), or if it is too high (e.g., 20-fold), no reversed ionic current (anti-gradient transport) can be observed (Fig. 2a).

The photo-induced active ion transport can be further confirmed by directly measuring the changes in ionic concentration in the two reservoirs via inductively coupled plasma optical emission spectroscopy (ICP-OES, Supplementary Note 8). For example, under a 10-fold concentration gradient, after light illumination for 180 s, K^+^ concentration in the high-concentration reservoir (*C*_H_) increases, while that in the low-concentration reservoir (*C*_L_) declines (Fig. 2b and Supplementary Table 4), showing anti-gradient transport. The K^+^ pumping rate increases with the light intensity, and approaches 0.78 ± 0.04 mol h^−1^ m^−2^ with light intensity of 120 mW cm^−2^. It also depends on the illumination position (Supplementary Note 9 and Supplementary Fig. 14) and the surface charge density of the GOM (Supplementary Note 10 and Supplementary Fig. 15).

Prolonging the illumination time (Supplementary Note 11 and Supplementary Fig. 16) or enhancing the light intensity (Supplementary Note 12 and Supplementary Fig. 17) facilitates the anti-gradient ion transport. For a fixed illumination time of 30 s, we measured the photocurrent under a series of concentration gradients and with different light intensities (Fig. 2c). In the bottom-right part of the dashed line (*I*_diff_), the ion transport is downhill, dissipating the concentration difference. While, in the upper-left part of the dashed line, the ion transport is against the concentration gradient, showing ion-pumping effect. Under lower concentration gradient, the anti-gradient ion transport can be realized with lower light intensity. But under higher concentration gradient, the minimum light intensity for anti-gradient ion transport should exceed a threshold value and rise with the concentration gradient.

Verified by ICP measurements, the photo-induced ion pumping against 10-fold concentration gradient (10/1 μM) can be achieved with many types of mono- and divalent ions (Fig. 2d and Supplementary Tables 5–6). Although having larger hydration radius, the pumping rate for divalent ions can be as high as the monovalent ions. Additionally, for ionic species larger than 4.5 Å, no effective changes in ionic concentration were found in both reservoirs. The cut-off size is quantitatively in agreement with the diffusion-driven ion permeation experiments^9^, and in accord with the effective height of the GO nanochannels (~0.92 nm). Considering the cross-sectional area (0.045 mm^2^) and the length (15 mm) of the GOM, the estimated ion permeation rate via classical diffusion under a 10 μM concentration difference is ~10^−5^ mol h^−1^ m^−2^, which is about 5 orders of magnitude lower than the measured ion pumping rates over a broad concentration range of 1–100 μM (Supplementary Fig. 13).

### Working principle

**Fig. 3:**
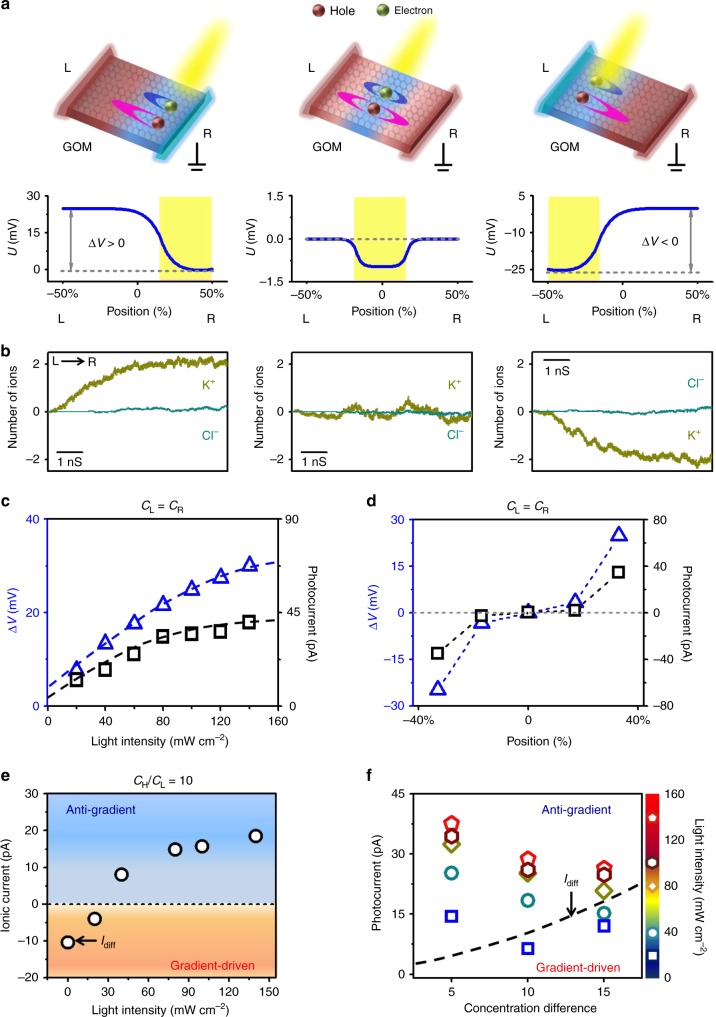
Mechanism. **a** Asymmetric diffusion and electromigration of electrons and holes result in the redistribution of electric potential along the GO strip and local low electric potential in the illuminated area. When illumination was on an off-center position (left and right), an electric potential difference (∆*V*) can be found. **b** MD simulations confirm the generation of horizontal cationic transport, depending on the illumination position. **c**, **d** The polarity and magnitude of ∆*V* and photocurrent depend on illumination position and light intensity. **e** Under 10-fold concentration gradient, photo-induced ion transport counterbalances diffusion current (*I*_diff_). Over a threshold light intensity, ionic current reverses to anti-gradient direction. **f** Similar trends were found under 5- to 15-fold concentration gradients. The threshold light intensity increases with the concentration gradient. The dashed line in (**f**) indicates *I*_diff_

The observed net ion transport directly correlates with a concomitant electric potential difference on GOM (Supplementary Note 13 and Supplementary Figs. 18–20)^26^. The photo-excited electrons and holes in the illuminated area would diffuse to the non-illuminated area driven by their concentration gradients (Supplementary Figs. 21–23). For GO-based materials, the diffusivity and mobility of holes are higher than that of the electrons (Supplementary Table 7). An electric potential difference is therefore established across the GOM via a diffusion-controlled charge separation^27^. We further develop a one-dimensional continuum model to quantify the mechanism (Supplementary Note 14)^28^. Asymmetric carrier diffusion in GO layers results in low electric potential in the illuminated area. If the illumination was applied to the central part, the electric potential distribution is symmetric and balanced between the two ends (Fig. 3a, middle). Otherwise, it leads to an electric potential difference (∆*V*) (Fig. 3a, left and right), whose polarity and magnitude depends on the illumination position and light intensity (Supplementary Figs. 24 and 25). From the model, one can see that Δ*V* is a direct consequence of asymmetric carrier diffusion. Meanwhile, carrier recombination facilitates the generation of Δ*V* (Supplementary Note 15 and Supplementary Fig. 26).

Furthermore, we applied the light-induced redistribution of the charge profile on a model GO nanochannel to elucidate the generation of net ionic flow with MD simulations (Supplementary Note 16). The lamellar nanochannel (length = 10.0 nm, width = 3.2 nm, height = 1.3 nm, Supplementary Fig. 27) connected two solution reservoirs. The initial ionic concentrations were equal (1.0 M) in both reservoirs. We find asymmetric light illumination in vertical direction induces a horizontal cationic flow through the lamellar nanochannel (Fig. 3b). The direction of the ionic flow depends on the illumination position, from the non-illuminated region to the illuminated region (Supplementary Fig. 28), in accord with the experimental observations (Fig. 1e). The K^+^ transport rate approaches 2.2 × 10^8^ ions s^−1^ with light intensity of 100 mW cm^−2^. Under light illumination, the GO nanochannel remains perfectly cation-selective (*t*_+_ → 1, Supplementary Fig. 29). A positive correlation is found between the photocurrent and the surface charge density on GO sheets. The model is able to reproduce the above experimental observations. Both the photo-induced electric potential difference and the ionic current highly depend on the illumination position and light intensity (Fig. 3c, d). Under concentration gradient, the light-induced ion transport can counterbalance the diffusion current (Fig. 3e, f). Beyond a threshold light intensity, the ionic flow can be reversed against the concentration gradient. Detailed model parameters are summarized in Supplementary Note 17 and Supplementary Tables 8–9.

Furthermore, we experimentally applied an electric potential distribution along the GOM to simulate the influence of light, and confirm the generation of a net ionic current (Supplementary Note 18 and Supplementary Figs. 30–31). To achieve long-range transport, inter-sheet carrier hopping should be considered (Supplementary Note 19). While the multi-sheet model gives a more precise description to the inter-sheet carrier transport and refines the electric potential profile, both models render an excellent support to the experimental observations (Supplementary Fig. 32). The main conclusions in Fig. 3 are unaffected. More intriguingly, we find that, under a transmembrane concentration difference of 10^−5^ M, the predicted ion pumping rate from the simplified model can be also 5 orders of magnitude higher than the estimated ion permeation rate from classical diffusion (Supplementary Note 20, Supplementary Fig. 33, and Supplementary Table 10).

## Discussion

**Fig. 4:**
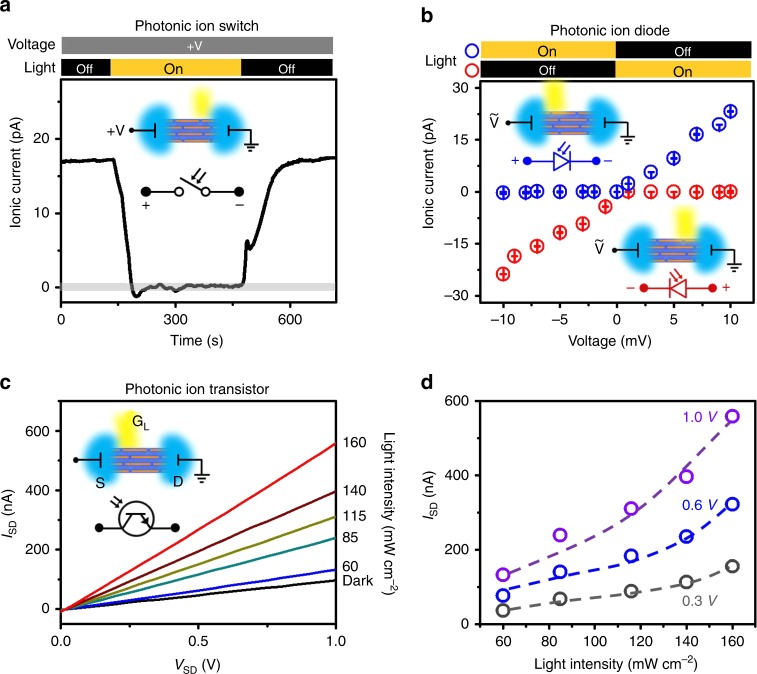
Photonic nanofluidic devices. **a** Photonic ion switch (PIS). Asymmetric light illumination (~150 mW cm^−2^) on GO strip perfectly switches off the ion transport driven by an electric voltage (~7 mV). **b** Photonic ion diode (PID). Under alternating electric field, photo-induced active ion transport blocks the ionic current at one voltage polarity with ultrahigh rectification ratio up to 10^4^. The polarity of PID depends on the illumination position. Error bars denote standard deviation. **c** Photonic ion transistor (PIT). Current–voltage (*I*_SD_–*V*_SD_) curves in dark and under light illumination. Light illumination is used as a photo-gate to control the ionic conductance between source and drain electrodes. **d** Transfer characteristics at *V*_SD_ of 0.3, 0.6, and 1.0 V. The electrolyte solutions were 1 μM KCl in (**a**)–(**d**)

Following this mechanism, we develop three types of fundamental elements for light-controlled nanofluidic circuits, termed PIS, PID, and PIT. As shown in Fig. 4a, upon asymmetric illumination, the photo-induced potential difference can be used to counterbalance an electric voltage in the same direction, and thereby blocks the transmembrane ionic current, forming a perfect switch-off state. The on–off ratio approaches 10^3^–10^4^. By synergistically operating the light and the alternating electric field, one can use the asymmetric light illumination to block the ionic current at only desired voltage polarity (Fig. 4b, Supplementary Note 21, and Supplementary Fig. 34), forming a highly efficient PID with rectification ratio up to ~10^4^. The polarity of the PID can be altered by changing the illumination position. Moreover, the localized low potential in the illuminated area can be treated as a photo-induced gate potential to control the horizontal ionic conductance^29^, functioning as a PIT (Fig. 4c). From the output characteristics and transfer curves (Fig. 4d), the source-drain current (*I*_SD_) increases with light intensity. The PIT shows p-type gating behavior. The photoresponsivity reaches up to 6.4 μA W^−1^ (Supplementary Fig. 35).

The generation of photocurrent and electric potential difference across the GOM is almost instantaneous upon light illumination. But, it takes time to reach steady state (Supplementary Note 22 and Supplementary Fig. 36). Due to the finite thickness of the GOM, the intensity of light irradiation in the depth direction was not homogeneous. Besides, to establish a steady-state electric potential distribution upon light irradiation also depends on the disorder of the GO assemblies^30^, as well as the intra- and inter-sheet charge traps^31^. This complex process may need more time to reach the equilibrium state. Similar results were found in previous photoconductivity measurements on macroscopic rGO membranes under continuous light illumination^32,33^. The time scale approaches several tens of seconds.

The photo-induced directional ion transport in 2D layered materials provides a new way for remote, non-invasive, and active control of the transport behaviors in synthetic systems. Sub-nanometer scale integration of atomically-thin 2D materials enables fabrication of highly compact nanofluidic architectures with extraordinarily high ionic pumping rates. By doping the 2D nano-building-blocks with photosensitive elements or molecules, their photo-responsiveness can be further extended for scalable and more precise applications in, for example, active ionic sieving, artificial photosynthesis, and modular nanofluidic computation.

## Methods

### Device fabrication

GOMs were prepared by vacuum filtration and stabilized via a thermal annealing process^22^. As schematically shown in Fig. 1a, the GO strip was top-sealed with a piece of transparent PDMS elastomer in a two-compartment electrochemical cell (made of Teflon) to avoid the leak of solution. Afterwards, the two ends of the sealed GO strip were trimmed off to connect with the solution reservoirs on the two sides. 3.5 ml ionic solution was filled in each reservoir. A pair of Ag/AgCl electrodes was used to record the transmembrane ionic current.

### Electrical measurements

Ionic current signals were recorded by a Keithley 2636B source meter. No externally applied voltage was needed, otherwise specifically mentioned. Light illumination was generated from a xenon lamp (Perfectlight CHF-500W). A transparent window was applied above the GOM to select the illumination position. The actual change in cation concentration in each reservoir was analyzed by ICP-OES to determine the ion transport rate (Supplementary Note 8).

### Theoretical methods

A one-dimensional continuity model was adopted to calculate the light-induced electric potential redistribution on GO surface, involving time-dependent evolution of electric potential and carrier densities. Photo-excitation, recombination, diffusion, and electro-migration were considered in the model. The partial differential equations were numerically integrated with a time step of 0.1 ps. Steady distribution was achieved once the maximum relative change of carrier density was less than 10^−9^. More details can be found in Supplementary Note 14.

MD simulation was conducted by NAMD package^34^. A 1.3-nm-height GO nanochannel was placed between two solution reservoirs. The system dimensions were 28.8 nm × 3.2 nm × 5.5 nm. The initial surface charge density was −25 mC m^−2^ and uniformly distributed. The charge density distribution calculated from the above model was then assigned to the MD model sheet to calculate the light-induced redistribution of electric potential. The system was equilibrated in isothermal–isobaric (NPT) ensemble under one atmosphere at 300 K. Then, the simulation was conducted in canonical (NVT) ensemble for data collection. More details can be found in Supplementary Note 16.

## Supplementary information

Supplementary Information

## Data Availability

The authors declare that the main data supporting the findings of this study are contained within the paper. All other relevant data are available from the corresponding author upon reasonable request.
