# Supplementary material for: Photo-induced ultrafast active ion transport through graphene oxide membranes
Source: Nat Commun. 2019 Mar 12;10:1171. doi: 10.1038/s41467-019-09178-x (PMC6414642; doi:10.1038/s41467-019-09178-x)
Supplement: Supplementary file 1 — Supplementary Information [file 41467_2019_9178_MOESM1_ESM.pdf]

Supplementary Information

Photo-induced ultrafast active ion transport through graphene oxide membranes

Jinlei Yang,[1,4] Xiaoyu Hu,[2] Xian Kong,[2] Pan Jia,[1,4] Danyan Ji,[1,4] Di Quan,[1,4] Lili Wang,[1] Qi Wen,[1] Diannan Lu,[2*] Jianzhong Wu,[2,3] Lei Jiang,[1] Wei Guo[1*]

[1] CAS Key Laboratory of Bio-inspired Materials and Interfacial Science, Technical Institute of Physics and Chemistry, Chinese Academy of Sciences, Beijing 100190, P. R. China.

[2] State Key Joint Laboratory of Chemical Engineering, Department of Chemical Engineering, Tsinghua University, Beijing 100084, P. R. China.

[3] Department of Chemical and Environmental Engineering, University of California, Riverside, California 92521, United States.

[4] University of Chinese Academy of Sciences, Beijing 100049, P. R. China.

* Correspondence to: wguo@iccas.ac.cn (W. G); ludiannan@tsinghua.edu.cn (D. L)

This PDF file includes:

Supplementary Notes 1-22

Supplementary Figures 1-36

Supplementary Tables 1-10

Supplementary References

Table of contents

Supplementary Note 1: XRD analysis.

Supplementary Note 2: Cation transference number.

Supplementary Note 3: Wavelength-dependent photocurrent and IPCE.

Supplementary Note 4: Temperature effect.

Supplementary Note 5: Characterizations on GOM upon light illumination.

Supplementary Note 6: Photo-response in other 2D layered materials.

Supplementary Note 7: Photo-response in control membrane samples.

Supplementary Note 8: ICP-OES analysis.

Supplementary Note 9: Ion pumping rate depends on light illumination position.

Supplementary Note 10: Influence of surface charge density.

Supplementary Note 11: Influence of illumination time.

Supplementary Note 12: Influence of light intensity.

Supplementary Note 13. Experimental evidences for diffusion-controlled charge separation mechanism.

Supplementary Note 14: Theoretical description of light-induced electric potential redistribution along GOM.

Supplementary Note 15. The role of carrier recombination.

Supplementary Note 16: MD simulations of photo-induced active ion transport through GO nanochannel.

Supplementary Note 17: Parameters for model calculation and MD simulations.

Supplementary Note 18: Experimental justification of theoretical predictions.

Supplementary Note 19: Consider carrier hopping in the model.

Supplementary Note 20: Compare ion pumping rate with classical diffusion.

Supplementary Note 21: Photo-modulation of ionic current.

Supplementary Note 22. The equilibrium time.

Supplementary References.

Supplementary Note 1. XRD analysis.

After being soaked in water, the layered structure is well preserved, revealed by XRD analysis (Supplementary Fig. 1). The *d*-spacing increases from about 0.77 to 1.26 nm due to the adsorption of additional water layers on GO sheets [1].

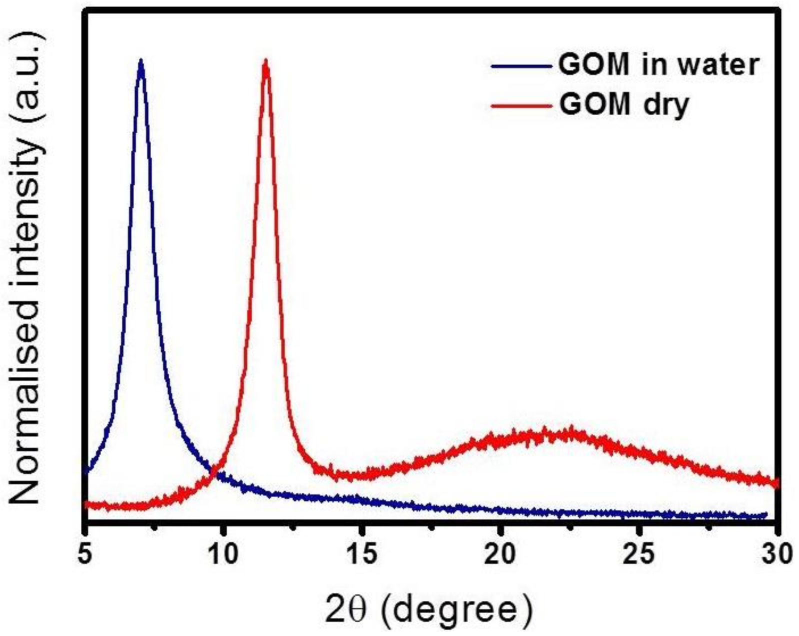

Supplementary Fig. 1. XRD analysis on GOMs before and after being soaked in water.

Supplementary Note 2. Cation transference number.

In the presence of transmembrane concentration difference, agar-saturated potassium chloride salt bridges were used to eliminate the imbalanced redox potential on the electrode|electrolyte interface. Ag/AgCl electrodes were used to record the current-voltage response with a Keithley 2636B source meter (Keithley Instruments). The diffusion potential were measured under different concentration gradients from 0 to 100-fold.

The cation transference number (t_+) quantifies the selective ion transportation. The value of t_+ can be calculated by [2]

$$2t_+ - 1 = \frac{E_{Diff}}{\frac{RT}{zF}\ln(\frac{\gamma_H c_H}{\gamma_L c_L})} \tag{1}$$

where R, T, z, F, γ, C_H, and C_L represent the gas constant, temperature, charge valent, Faraday constant, activity coefficient of ions, high and low ion concentrations, respectively. Due to the presence of negative surface charge on GO sheets and the extremely narrow channel width, the measured t_+ is over 0.97 (Supplementary Fig. 2). The results were summarized in Supplementary Table 1.

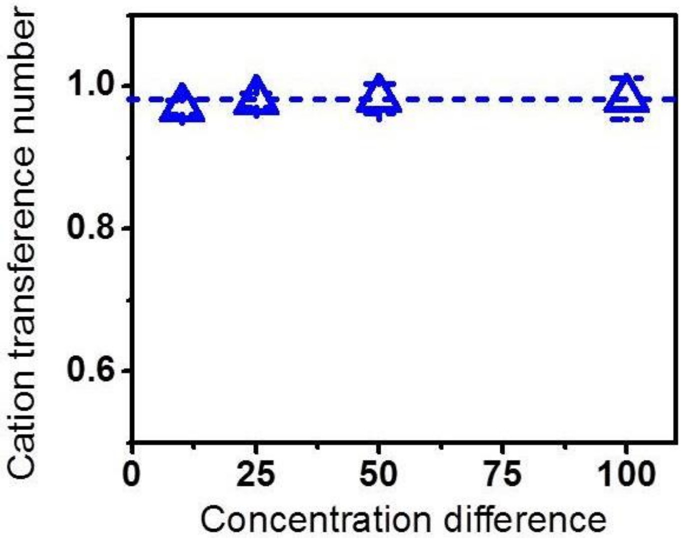

Supplementary Fig. 2. Cation transference number (t_+) measured under varied concentration differences. Error bars denote standard deviation.

Supplementary Table 1. The activity coefficient (γ), diffusion potential, and cation transference number (t_+).

Concentration gradient (M/M)	γ	Diffusion potential (mV)	t_+
$10^{-6}/10^{-5}$	0.999/0.996	55.3 ± 1.5	0.97 ± 0.01
$10^{-6}/2.5\times10^{-5}$	0.999/0.994	79.7 ± 3.2	0.98 ± 0.01
$10^{-6}/5\times10^{-5}$	0.999/0.992	97.3 ± 5.5	0.98 ± 0.02
$10^{-6}/10^{-4}$	0.999/0.989	108.0 ± 2.7	0.98 ± 0.02

Supplementary Note 3. Wavelength-dependent photocurrent and IPCE.

The relationship between the photocurrent and the wavelength of the incident light were investigated by separately applying a series of optical filters with transmission wavelengths centered at 405-, 450-, 475-, 520-, 550-, 600-, 650-, 700-nm, and infrared region (800-1100 nm) in front of the simulated sunlight. The light beam was shed on the right 1/3 of the GO strip, and the light intensity at each wavelength was 20 mW cm^{-2}. UV-vis spectrophotometer (UV-2600, SHIMADZU) was employed to measure the absorption spectrum of GO dispersion (0.33 mg ml^{-1}).

The incident photon to current efficiency (IPCE) was defined as [3],

$$\eta = \frac{P_E}{P_L} \times 100\% \quad (2)$$

$$P_E = \int I \cdot dV \quad (3)$$

where P_E was the generated electric power, P_L was the incident light power, I and V were the current and voltage obtained from the scanning current-voltage curve (Supplementary Fig. 3A). Upon light illumination, non-zero intercepts on both the current and voltage axes were observed showing the generation of net ion transport from the device.

The resulting electric power density and the IPCE at each wavelength generally agree with the absorption spectrum of GO dispersion (Supplementary Fig. 3B and 3C). The maximum IPCE was found at wavelength of 405-nm, which is on the order of ~10^{-6}% in 1 μM KCl solution. This value can be further enhanced by nearly an order of magnitude by using high-concentration electrolyte solutions (Supplementary Fig. 3D). The IPCE found here is largely lower than the solid-state photoelectric devices. One possible reason is that, in our device, the energy of light is used to power the transport of ionic species in aqueous solution. The mass of ions is much larger, and their mobility in aqueous solution is much lower, than the charge carriers (electrons and holes) in solid-state photoelectric materials. In addition, in previous light-driven ion transport systems [4,5], the ion transport and the light illumination were conducted in the same direction, perpendicular to the membrane surface. In contrast, in the present

work, the ion transport goes in the horizontal direction, whereas the light illumination is from the vertical direction (Fig. 1d). The ion transport pathway in our work approaches centimeter-long, which is much longer than that in previous works (several nanometers in lipid bilayer or tens of microns in porous polymeric membrane). These reasons may limit the IPCE.

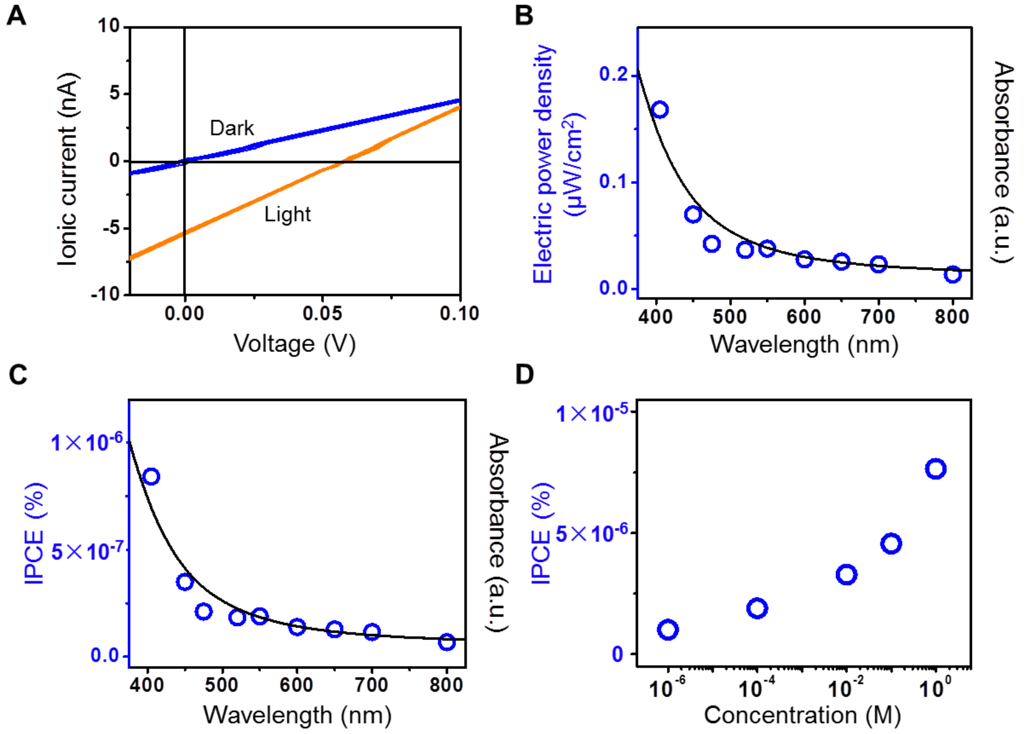

Supplementary Fig. 3. Evaluation of IPCE. (A) Typical current-voltage curves in dark and upon light illumination (wavelength ~405 nm). (B and C) Electric power density and corresponding IPCE with respect to the light wavelength. The electrolyte solution was KCl (1 μM). The light intensity at each wavelength was 20 mW cm^{-2}. The absorption spectrum of GO dispersion was also shown. (D) IPCE with respect to the electrolyte concentration. The wavelength was centered at 405 nm.

Supplementary Note 4. Temperature effect.

We measured the temperature change on the GOM surface by infrared radiation thermal imaging (IRS S6). The GO strip becomes heated upon light illumination relative to the surrounding area (Supplementary Fig. 4A and 4B). The temperature increase (ΔT) shows positive correlation with the light intensity and the illumination time. Under a typical light intensity of 50 mW cm^{-2}, the temperature increase is no more than 11 ^{o}C after light illumination for about 30 seconds.

Then, we establish a local high-temperature area on the GO strip by a ceramic heater to simulate the photothermal effect, and check the influence of the temperature to the ion transport property. The heater was separately applied on the left, middle, and right part of the GO strip. The heating process lasted for about 30 seconds to reach the peak ΔT of about 5, 10, and 20 ^{o}C, respectively. Afterwards, the testing devices were naturally cooled in air. Other experimental conditions for the electrochemical measurements were identical with that mentioned in the main text. As shown in Supplementary Fig. 4C, in both the heating and cooling processes, no net ionic current was observed.

We also note that, recently, high-temperature reduced graphene oxide nanosheets were discovered as an effective thermoelectric conversion material [6]. However, in these experiments, they used very high temperature (~ 3000 K) to reduce GO, in order to make it highly conductive. The working temperature difference was also as high as several thousand K. However, in our experiments, the temperature rise was typically less than 20 ^{o}C. The light-induced reduction of GO is very limited (see Supplementary Note 5 below). The thermoelectric property is therefore negligible. These evidences lead us to believe that the temperature effect is insignificant at conditions considered in this work.

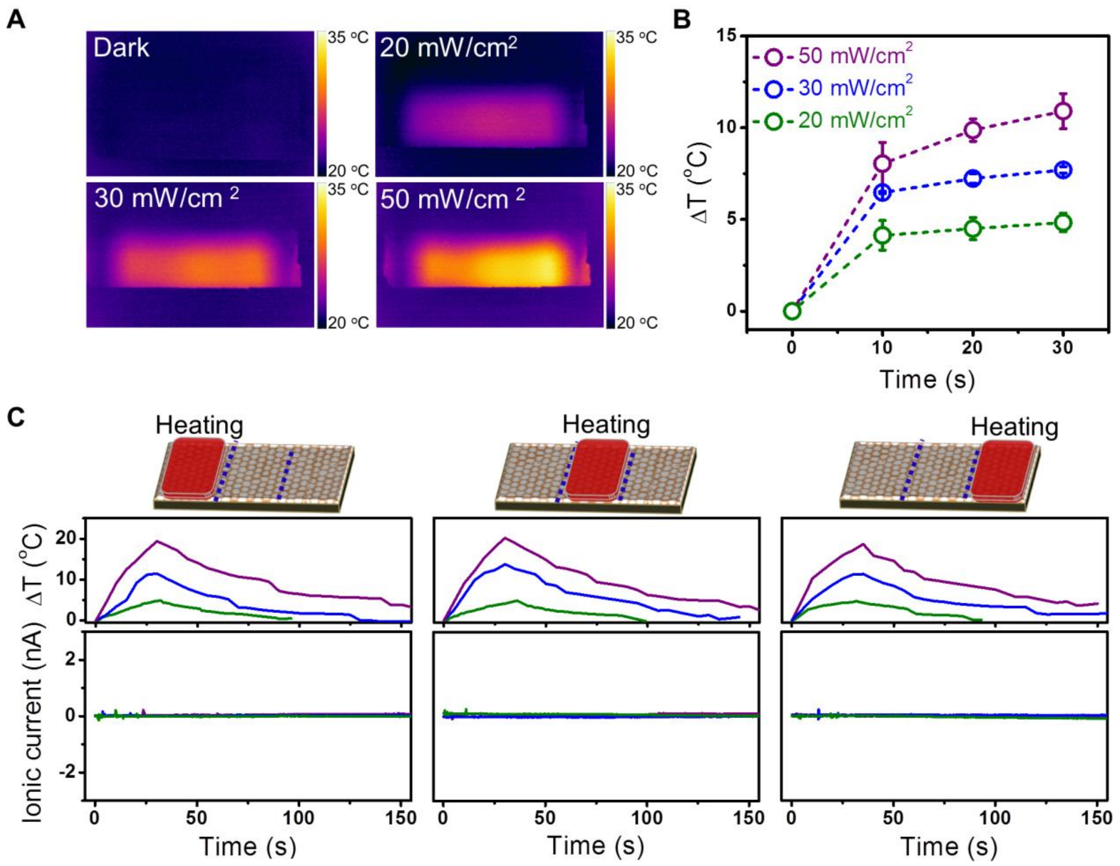

Supplementary Fig. 4. Temperature effect. (A) Thermal images of the GO strip before and after light illumination for 30 seconds. The light intensity was 20, 30, and 50 mW cm^{-2}, respectively. (B) Temperature increase (ΔT) as a function of illumination time under different light intensities. Error bars denote standard deviation. (C) Time traces of ionic current in controlled heating and cooling process. A ceramic heater was attached separately on the left, middle, or right part of the GO strip. No net ionic current was observed in all cases.

To determine the influence of temperature, we further measured the photocurrent and the photovoltage at temperatures from 4 to 40 ^{o}C. In the testing temperature range, the photocurrent shows weak dependence on temperature (Supplementary Fig. 5A). In contrast, the photovoltage shows a notable decrease with the increasing temperature (Supplementary Fig. 5B). The electric potential difference generates from the asymmetric carrier diffusion between the photo-excited electrons and holes (see the discussions in Supplementary Note 14). In GO, the diffusivity and mobility of holes is

higher than that of electrons. With the rise in temperature from 4 to 40 °C, the mobility of the electrons increases faster than that of the holes [7], which narrows the difference between them at high temperatures. Therefore, the diffusion-controlled charge separation process generates lower electric potential difference at high temperatures. In addition, the ionic conductance through the GOM increases with the temperature (Supplementary Fig. 5C). These evidences explain why the photocurrent shows weak temperature dependence in this range (Supplementary Fig. 5A). The experimental results can be well supported by theoretical calculations in the same temperature range (Supplementary Figs. 5D-5F). The temperature-dependent model parameters were obtained from ref. 7. Other parameters were identical with that used in Supplementary Note 14 and Supplementary Note 15.

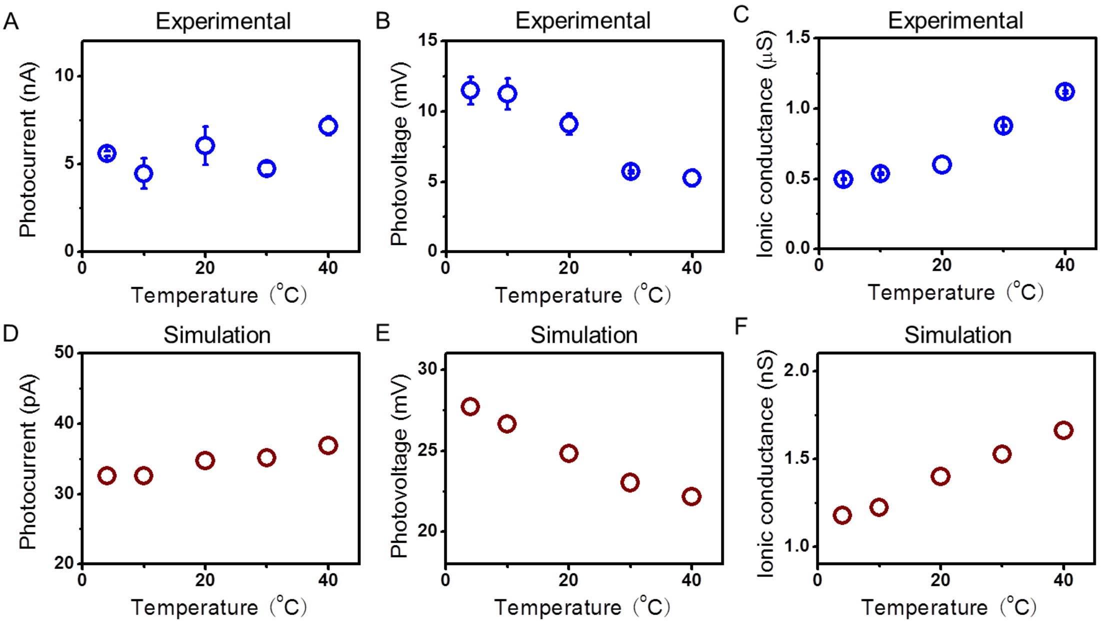

Supplementary Fig. 5. The influence of temperature on photocurrent, photovoltage, and ionic conductance through GOM from 4 to 40 °C. (A-C) Experimental results. (D-F) Theoretical predictions. Error bars denote standard deviation.

Supplementary Note 5. Characterizations on GOM upon light illumination.

We used UV-vis absorption spectrum, Raman spectrum, Fourier transform infrared (FTIR) spectrum, and X-ray photoelectron spectroscopy (XPS) to characterize the surface functional groups and the oxidation state; and used the surface contact angle measurement to determine the surface hydrophilicity before and after light illumination.

To avoid the discrepancy between samples, the UV-vis absorption spectrum measurements (SHIMADZU, UV-2600) were carried out on the same GOM, irradiated with visible light for 0, 10, 20, and 30 seconds. The light intensity was 100 mW cm^{-2}. As shown in Supplementary Fig. 6, the GOM shows a broad absorption band with a long tail down to 900 nm in the UV-vis spectrum. The peak at 227 nm was assigned to the π-π* transition, and the shoulder at about 300 nm was assigned to the n-π* transition of C=O [8]. However, the four absorption curves for different light illumination time superpose with each other. No observable change in the absorption intensity, or in the peak shift, can be identified upon light illumination.

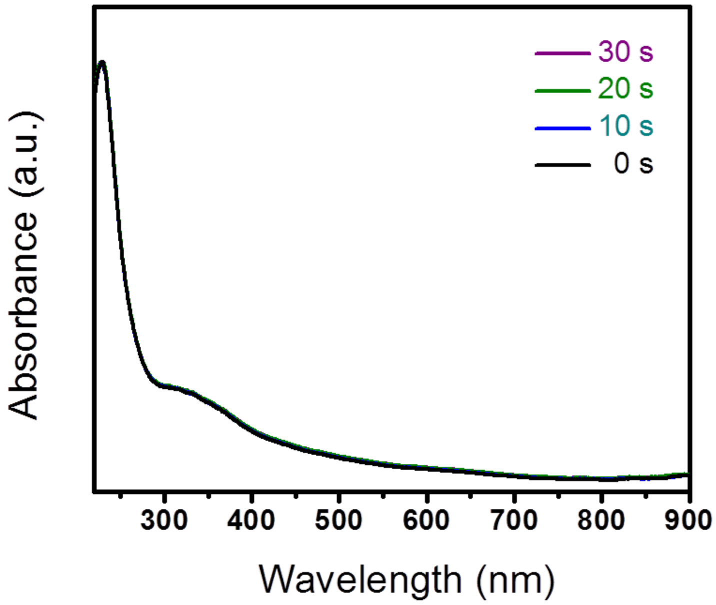

Supplementary Fig. 6. UV-Vis absorption spectra of GOM before and after light illumination for 10, 20, and 30 seconds. The light intensity was 100 mW cm^{-2}. The absorption curves for different illumination time superpose with each other.

Raman spectrum (Horiba, Labram HR Evolution) were used to determine the oxidation state before and after light illumination. The intensity ratio of D and G bands at ~1350 cm^{-1} and ~1580 cm^{-1} (I_D/I_G), was used as a metric of the rise in the content of sp^2 domains upon reduction [9,10]. The sample treatment was identical with that used for the UV-vis absorption spectrum measurements. As shown in Supplementary Fig. 7 and Supplementary Table 2, within the illumination time of 30 seconds, I_D/I_G gradually increases from 0.97 to 1.03, indicating a weak reduction upon light illumination.

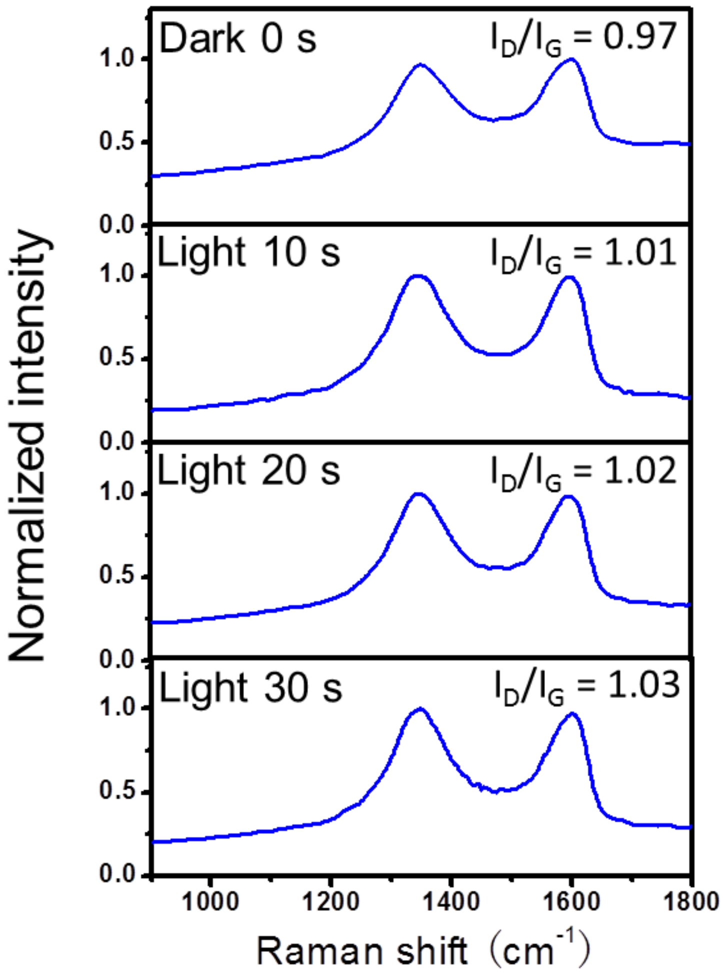

Supplementary Fig. 7. Raman Spectra of GOM before and after light illumination for 10, 20, and 30 seconds. The intensity ratio of D and G peak (I_D/I_G) was calculated. The light intensity was 100 mW cm^{-2}.

Supplementary Table 2. Summary of the Raman, FTIR, and XPS characterizations before and after light illumination.

Sample	Raman ratio I_D/I_G[a]	FITR content of C=C band (%)[b]	XPS C/O atomic ratio[c]
non-illuminated GOM	0.97	41.62	2.09
Light 10 s	1.01	42.57	2.14
Light 20 s	1.02	42.82	2.15
Light 30 s	1.03	43.13	2.19

[a] Intensity of D band divided by the intensity of the G band.
[b] Area of C=C band divided by the sum of the areas of C=O and C=C bands.
[c] Area of C1s peak divided by the area of O1s peak.

FTIR spectrum (Bruker, TENSOR-27) was used to characterize the variation of the surface functional groups before and after light illumination. The sample treatment was identical with that used for the UV-vis absorption spectrum measurements. There is no frequency shift in the band position corresponding to the functional groups, such as –OH (~3200 cm^{-1}), C=O (1718 cm^{-1}), C=C (1618 cm^{-1}), and C-O-C (1045 cm^{-1}) (Supplementary Fig. 8). The reduction extent can be evaluated from the relative area of C=C band divided by the sum of the areas of C=O and C=C bands [9]. As summarized in Supplementary Table 2, in the non-illuminated GOM, the C=C bands accounts for 41.62%. After light illumination for 30 seconds, this value gradually increases to about 43.13% due to the progressive oxygen removal, suggesting a very small amount of reduction.

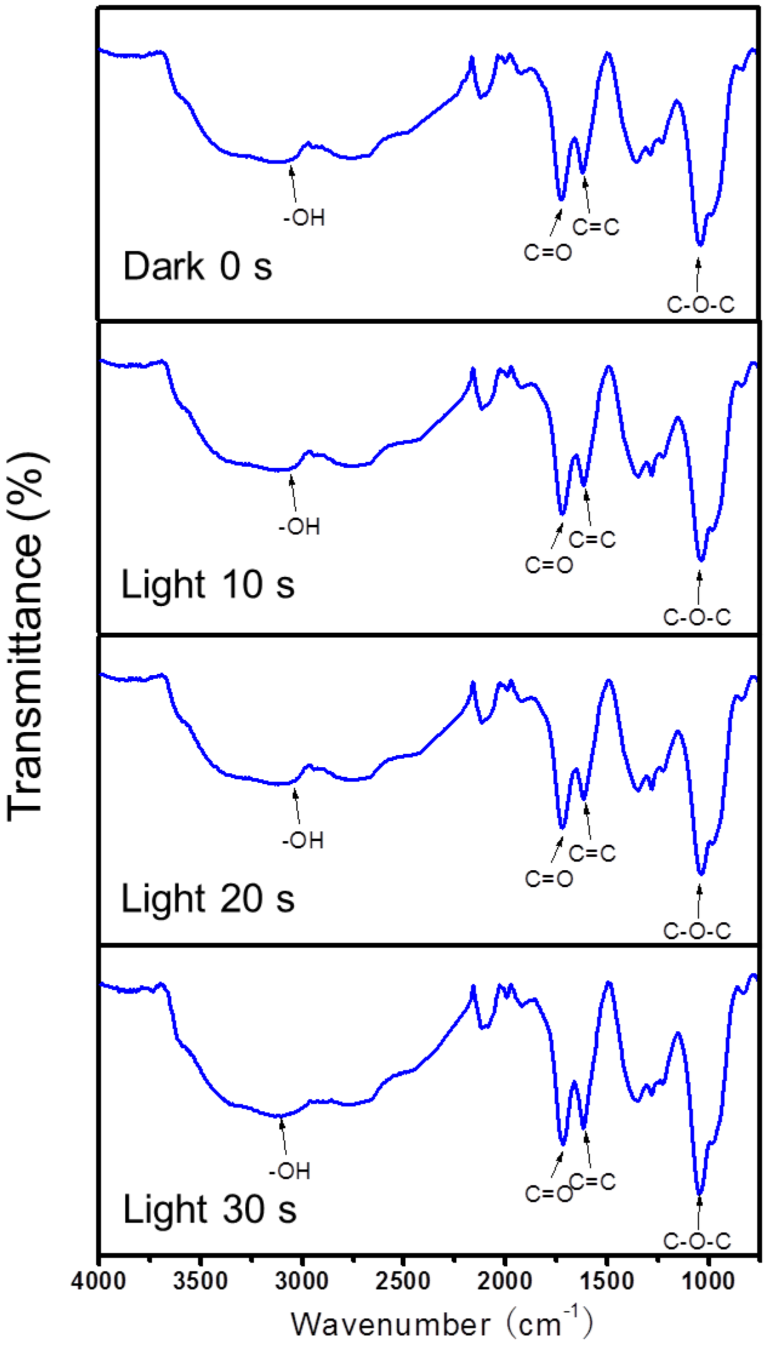

Supplementary Fig. 8. FTIR spectra of GOM before and after light illumination for 10, 20, and 30 seconds. The light intensity was 100 mW cm^{-2}.

XPS characterizations (TF Scientific, ESCALAB 250Xi) were further used to determine the reduction extent and variation of functional groups upon light illumination. The tests were carried out on separate samples, which were kept in dark and illuminated by light (100 mW cm^{-2}) for 10, 20, and 30 seconds. The results were shown in Supplementary Fig. 9 and Supplementary Table 2. Before light illumination, the C/O atomic ratio is 2.09. After light illumination for 30 seconds, the C/O atomic ratio gradually increases to 2.19, suggesting the progressive removal of oxygenated groups and restore of C=C on the basal plane. Due to the relatively weak light intensity and short illumination time, the reduction extent is very weak.

Furthermore, from the high resolution C-1s spectrum (Supplementary Fig. 9), the content of C=C/C-C (at 284.8 eV), C-O-C/C=O (at 286.9 eV), and O-C(=O) (at 288.5 eV) groups before and after light illumination can be characterized. The percentages of the functional groups in dark and illuminated conditions were summarized in Supplementary Table 3. The small increase in C=C groups and the slight decrease in oxygen-containing groups, such as C-O-C/C=O and O-C(=O), indicate a very weak reduction of the GOM after light illumination. In addition, the small amount decrement in the oxygen-containing groups also slightly reduces the surface charge density. But its influence is very limited. These results are in agreement with the Raman and FTIR characterizations.

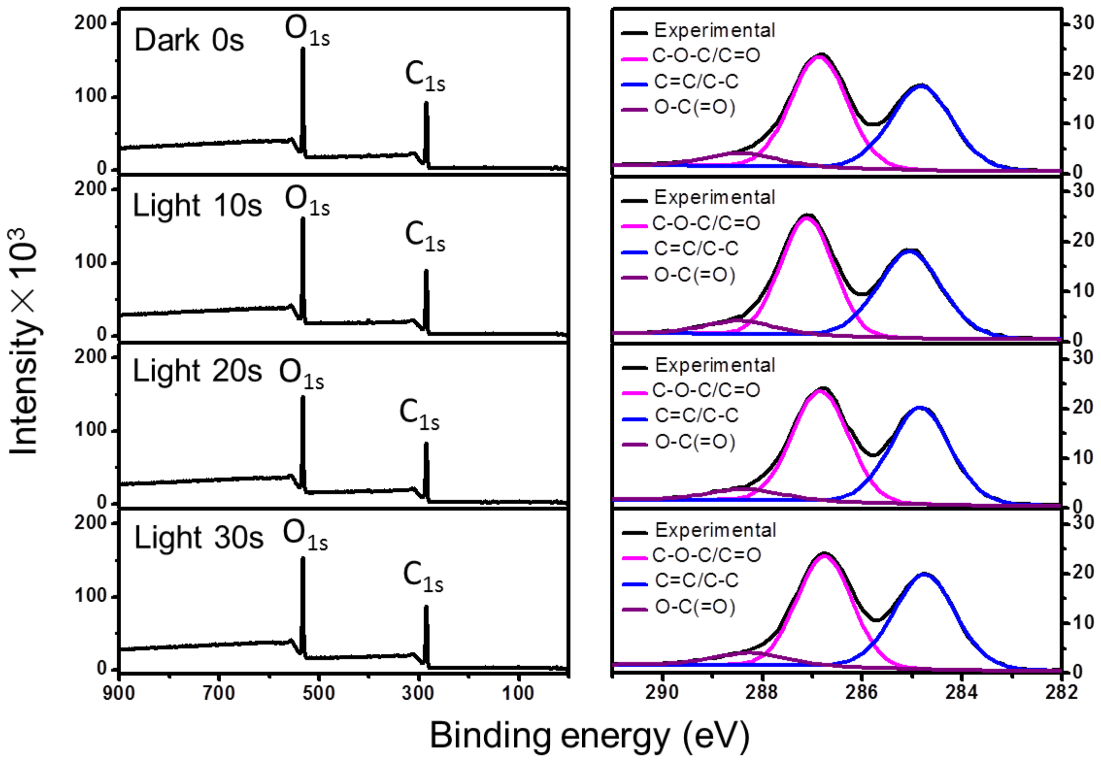

Supplementary Fig. 9. Survey (Left) and high resolution (Right) C1s XPS spectra of GOM before and after light illumination for 10, 20, and 30 seconds. The light intensity was 100 mW cm^{-2}.

Supplementary Table 3. XPS characterizations of the content of the functional groups before and after light illumination.

	C=C/C-C (%)	C-O-C/C=O (%)	O-C(=O) (%)
non-illuminated GOM	42.35	50.08	7.58
Light 10 s	44.29	48.22	7.49
Light 20 s	45.83	47.91	6.26
Light 30 s	46.93	47.18	5.89

Surface contact angle measurements (CA, Data Physics, OCA20) were carried out to characterize the variation in hydrophilicity before and after light illumination. The sample treatment was identical with that used for the XPS test. The light intensity was 100 mW cm^{-2}. A gradual increase in CA from about 49.0^o to 54.7^o was observed with respect to the prolonged illumination time (Supplementary Fig. 10). The GOM becomes slightly less hydrophilic after light illumination. This phenomenon results from the small amount of reduction upon light illumination, which is in accord with the Raman, FITR, and XPS characterizations.

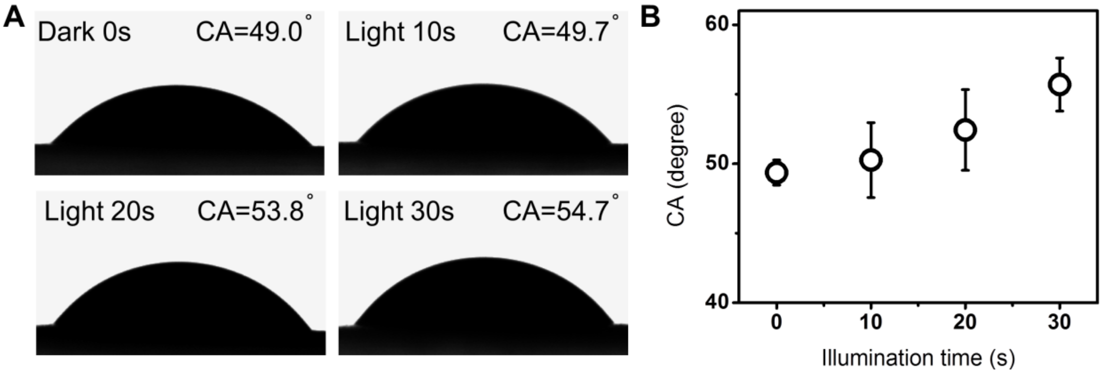

Supplementary Fig. 10. Surface contact angle characterizations on GOM before and after light illumination for 10, 20, and 30 seconds. The light intensity was 100 mW cm^{-2}. Error bars denote standard deviation.

Supplementary Note 6. Photo-response in other 2D layered materials.

Other 2D layered membranes were also fabricated via vacuum filtration of WSe_2, MoS_2, WS_2, and rGO nanosheets, respectively. Photocurrent measurements were carried out in identical conditions as that mentioned in the main text. Asymmetric light illumination also generates net ion transport horizontally through these membranes (Supplementary Fig. 11).

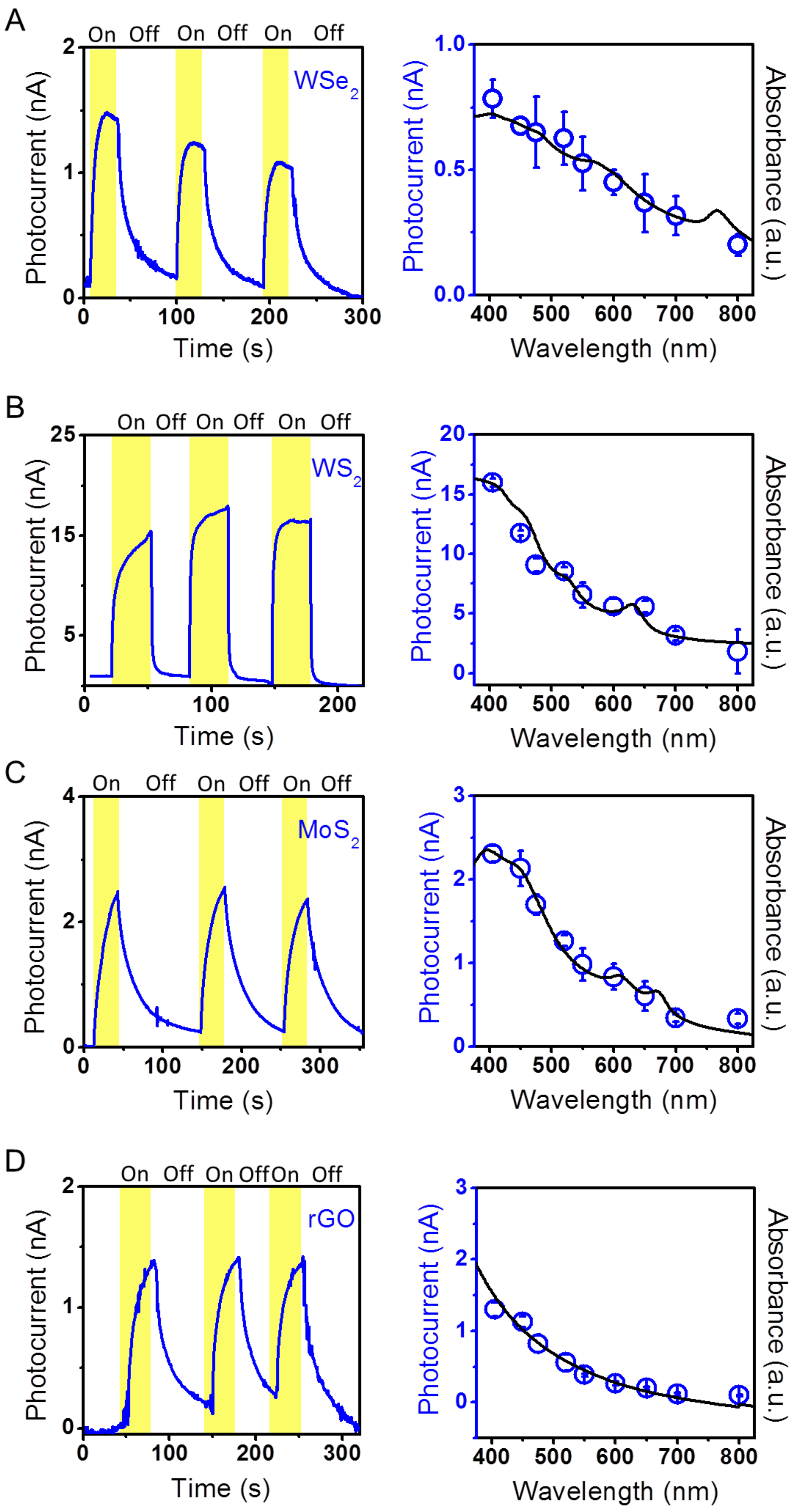

Supplementary Fig. 11. Generation of photocurrent and its wavelength dependence in other 2D layered materials, including WSe_2, MoS_2, WS_2, and rGO. The light intensity was 100 (left panels) and 20 mW cm^{-2} (right panels), respectively. KCl solution (1 μM) was used as the electrolyte. Error bars denote standard deviation.

Supplementary Note 7. Photo-response in control membrane samples.

We also checked the photo-response with pH test papers (Control 1#) and cellulose acetate filter membranes (Control 2#), separately, as control samples. In sharp contrast to the GOM, no measurable photo-response was found with these control membrane samples (Supplementary Fig. 12).

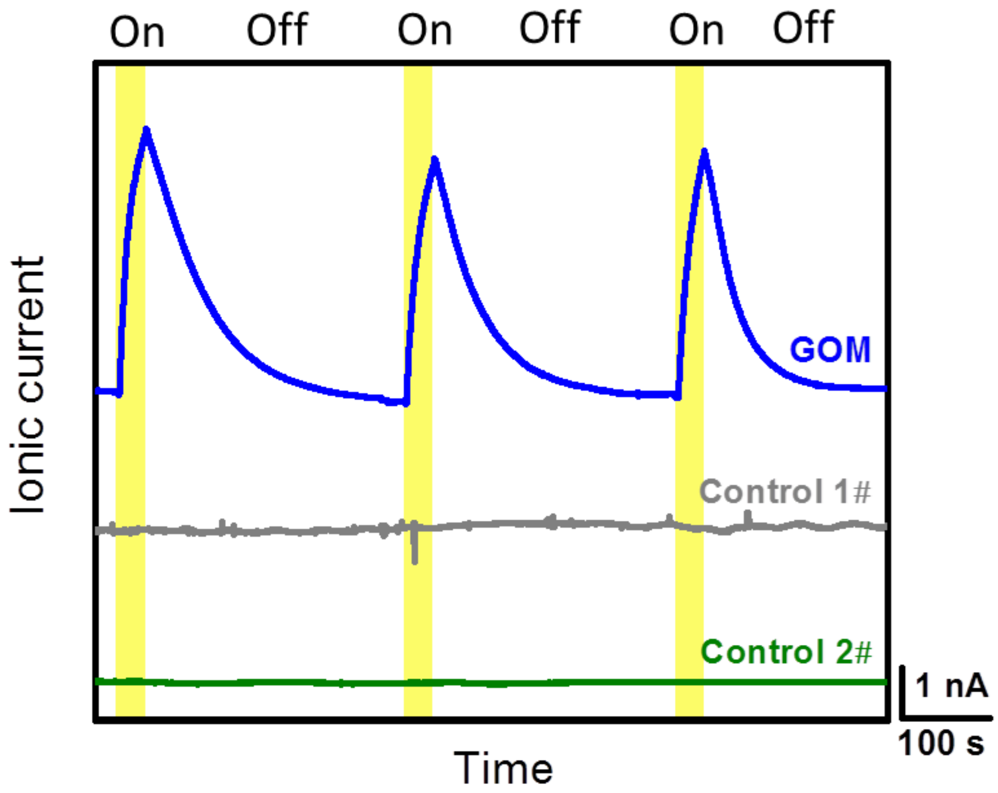

Supplementary Fig. 12. Photo-response in GOM and control membrane samples. Control 1#, pH test paper. Control 2#, cellulose acetate membrane. No measurable photo-response was found with control samples. The photocurrent was collected with light intensity of 100 mW cm^{-2} in 1 μM KCl.

Supplementary Note 8. ICP-OES analysis.

Inductively coupled plasma optical emission spectrometry (ICP-OES) was measured on a Varian 710-OES instrument to determine the ion concentrations. Samples were taken immediately from the two reservoirs before and right after light illumination for 180 seconds. Then, the variations in ion concentration were analyzed via ICP-OES, and the pumping rate was calculated accordingly. The actual concentration change measured from the two reservoirs could not be strictly equal. The pumping rate was calculated by averaging the concentration change from the two reservoirs.

For comparison, we estimate the ion permeation rate via classical diffusion (J_c) by[11]

$$J_c = D \times \Delta C \times A/L \tag{4}$$

where D is the bulk diffusion coefficient of ions in water, ΔC is the applied concentration gradient across the GOM (C_H = 10 μM and C_L = 1 μM, identical with the experiments), A is the total cross-sectional area of GOM (0.045 mm^2), L is the diffusion length through the GOM (15 mm).

Supplementary Table 4. ICP data for K^+ concentration in the two reservoirs before and after light illumination. The concentration change and corresponding pumping rate were shown in Fig. 2B.

Light intensity ($mW\ cm^{-2}$)	0	80	100	120
Low-concentration reservoir ($\mu g\ L^{-1}$)	25.89	20.98	16.13	5.52
High-concentration reservoir ($\mu g\ L^{-1}$)	276.34	280.30	284.95	294.94

Supplementary Table 5. ICP data for cation concentrations in the two reservoirs before and after light illumination. Corresponding ion pumping rates were summarized in Supplementary Table 6 and were plotted in Fig. 2d. The light intensity was 100 mW cm^{-2}.

	Low-concentration (μg L^{-1}) before/after	High-concentration (μg L^{-1}) before/after
K^+	56.30/48.76	574.31/585.70
Na^+	22.34/18.38	200.65/207.65
Pb^{2+}	134.09/99.39	1512.26/1598.95
Ni^{2+}	51.15/41.08	506.28/528.60
Ca^{2+}	125.70/72.70	410.86/422.93
Cu^{2+}	44.47/33.56	531.33/556.00
Co^{2+}	47.81/37.85	489.25/512.19
Cd^{2+}	103.01/79.03	1050.88/1096.54
Mg^{2+}	27.14/18.56	208.38/215.35
Mn^{2+}	48.22/43.40	509.15/519.93
Fe^{3+}	39.30/39.74	460.06/457.77
Cr^{3+}	45.01/45.47	525.01/525.98
$[Ru(bipy)_3]^{2+}$	75.15/75.32	920.12/919.09

Supplementary Table 6. Tested ionic species and their hydrated radius, bulk diffusion coefficient, experimentally measured pumping rates, and estimated ion permeation rate via classical diffusion.

	Hydrated radius (Å)	Diffusion coefficient ($cm^2\ s^{-1}$)	Pumping rate ($mol\ h^{-1}\ m^{-2}$)	Estimated permeation rate ($mol\ h^{-1}\ m^{-2}$)
K^+	3.31	2.00×10^{-5}	0.38	0.43×10^{-5}
Na^+	3.58	1.33×10^{-5}	0.37	0.29×10^{-5}
Pb^{2+}	4.01	0.95×10^{-5}	0.46	0.20×10^{-5}
Ni^{2+}	4.04	0.66×10^{-5}	0.42	0.14×10^{-5}
Ca^{2+}	4.12	0.79×10^{-5}	1.26	0.17×10^{-5}
Cu^{2+}	4.19	0.71×10^{-5}	0.44	0.15×10^{-5}
Co^{2+}	4.23	0.73×10^{-5}	0.44	0.16×10^{-5}
Cd^{2+}	4.26	0.72×10^{-5}	0.48	0.15×10^{-5}
Mg^{2+}	4.28	0.71×10^{-5}	0.51	0.15×10^{-5}
Mn^{2+}	4.38	0.71×10^{-5}	0.23	0.15×10^{-5}
Fe^{3+}	4.57	0.60×10^{-5}	Non-detectable	0.13×10^{-5}
Cr^{3+}	4.61	0.60×10^{-5}	Non-detectable	0.13×10^{-5}
$[Ru(bipy)_3]^{2+}$	5.90	/	Non-detectable	/

We further tested the ion pumping rate at different concentrations ranging from 1 to 100 μM (C_{low}). The transmembrane concentration difference was fixed at 10 μM (C_{high}-C_{low}=10 μM). Other experimental conditions were identical with that used for Fig. 2d. For ΔC=10 μM, the estimated ion permeation rate via classical diffusion was 0.48×10^{-5} mol h^{-1} m^{-2} (Supplementary Note 8). As shown in Supplementary Fig. 13, the ion pumping rate mildly increases with the ionic concentration. In the tested concentration range, the ratio of the ion pumping rate to the estimated ion permeation rate approaches five orders of magnitude.

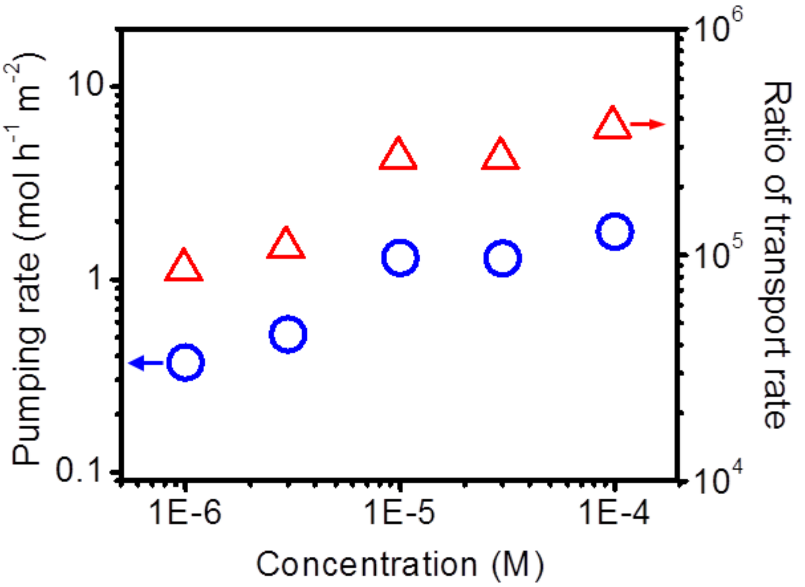

Supplementary Fig. 13. Ion pumping rate at different concentrations. The ionic concentration at the low-concentration side was 1, 3, 10, 30, 100 μM, respectively. The transmembrane concentration difference was 10 μM. Compared with the classical ion permeation rate, the ion pumping rate was five orders of magnitude higher.

Supplementary Note 9. Ion pumping rate depends on light illumination position.

As shown in Supplementary Fig. 14, we directly measured the changes in ionic concentration in the two reservoirs via ICP-OES analysis when the light illumination located separately on the left, middle, and right part of the GO strip. The light intensity was 100 mW cm^{-2}, and the illumination time was 180 seconds. When the light beam located on the on the left 1/3 of the GO strip (I), the K^+ concentration in the high-concentration reservoir (C_H) increases, while that in the low-concentration reservoir (C_L) declines, showing anti-gradient ion transport. The cation transfer rate was -0.51±0.06 mol h^{-1} m^{-2}. When the light beam located on the on the middle or right 1/3 of the GO strip (II and III), the K^+ concentration in the high-concentration reservoir (C_H) falls down, while that in the low-concentration reservoir (C_L) goes up, showing gradient-driven ion transport. Their cation transfer rates were 0.60±0.01 and 0.80±0.14 mol h^{-1} m^{-2}, respectively. These results show that the ion pumping rates also depend on the illumination position, in accord with the photocurrent (Fig. 1e and Fig. 2a).

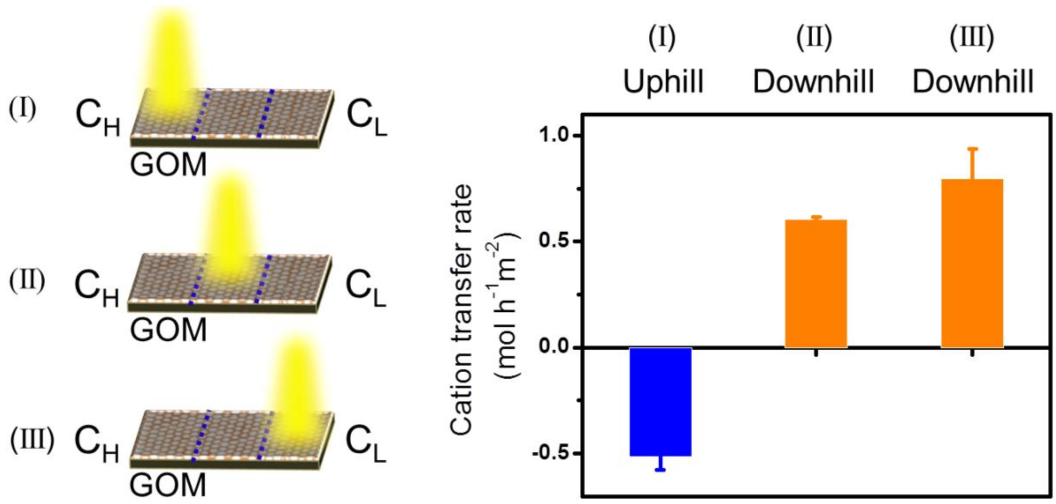

Supplementary Fig. 14. Cation transfer rate under a concentration gradient (C_H=10 μM, and C_L=2 μM). The light illumination was carried out on different positions. The light intensity was 100 mW cm^{-2}. The illumination time was 180 seconds. Error bars denote standard deviation.

Supplementary Note 10. Influence of surface charge density.

The surface charge density was adjusted by tuning the pH of the electrolyte solution [12]. The electrolyte solution was 10 μM KCl. To avoid the interference of protons and metal ions brought by the acid or alkaline solution, the test was conducted in the pH range from 6.0 to 8.0. The tests were conducted for three times on the same device. As shown in Supplementary Fig. 15, the cation transfer rate roughly goes up with the pH. This observation is in agreement with the simulation results shown in Supplementary Fig. 27. Enhanced surface charge density can promote the cation transfer rate.

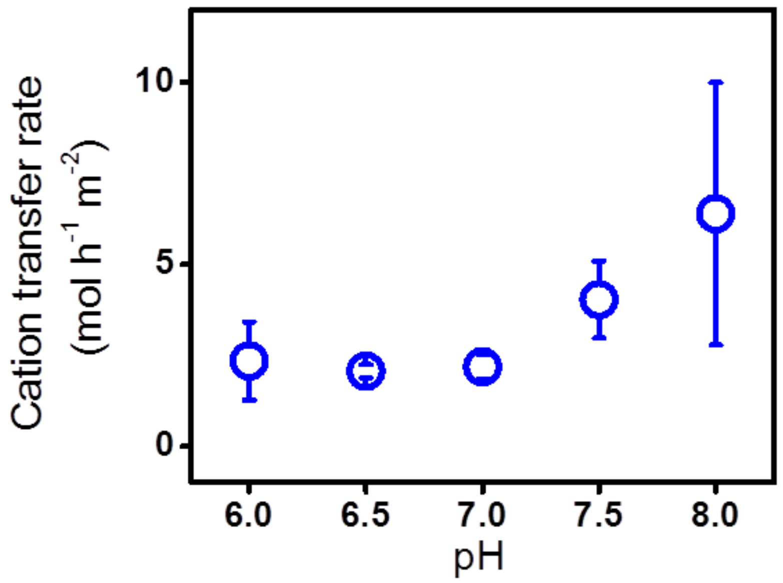

Supplementary Fig. 15. Cation transfer rate at varied pH. The light intensity was 100 mW cm^{-2}. The illumination time was 180 seconds. Error bars denote standard deviation.

Supplementary Note 11. Influence of illumination time.

With prolonged illumination time, the photocurrent continuously goes up (Supplementary Fig. 16A). Under transmembrane concentration gradient, with shorter illumination time (< 20 s), the generated photocurrent is smaller than the diffusion current (I_{diff}), and no ionic current reversion can be found (Supplementary Fig. 16B). With prolonged illumination time, the ionic current reverses to the opposite direction, showing anti-gradient ion transport.

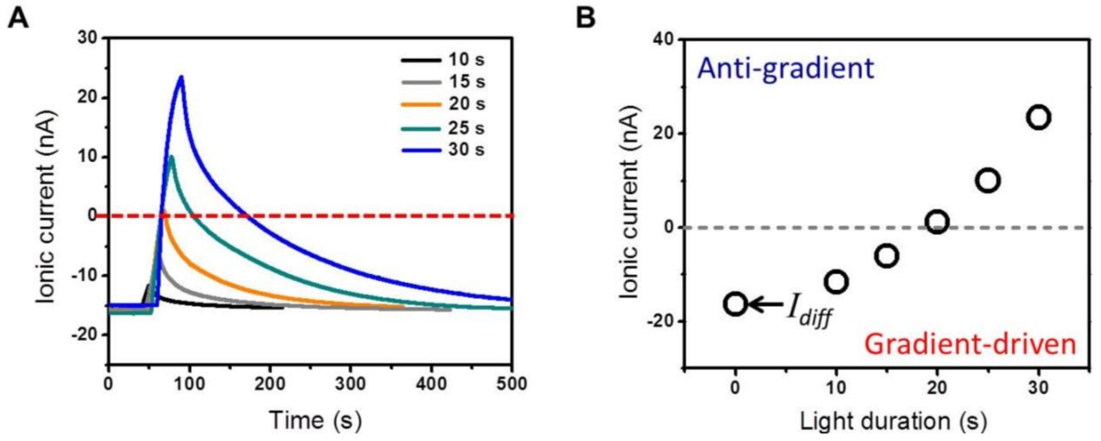

Supplementary Fig. 16. Influence of illumination time. **(A)** Time traces of the ionic current under asymmetric light illumination for different times. **(B)** Peak values of the ionic current with respect to the illumination time. The light intensity was 100 mW cm^{-2}. The concentration difference is C_{high}/C_{low}=10 μM/1 μM.

Supplementary Note 12. Influence of light intensity.

The generated photocurrent goes up with enhanced light intensity (Supplementary Fig. 17A). Under transmembrane concentration gradient, with lower light intensity (< 50 mW cm^{-2}), the generated photocurrent is smaller than the diffusion current (I_{diff}, Supplementary Fig. 17B), and no ionic current reversion can be found. With enhanced light intensity, the ionic current reverses to the opposite direction, showing anti-gradient ion transport.

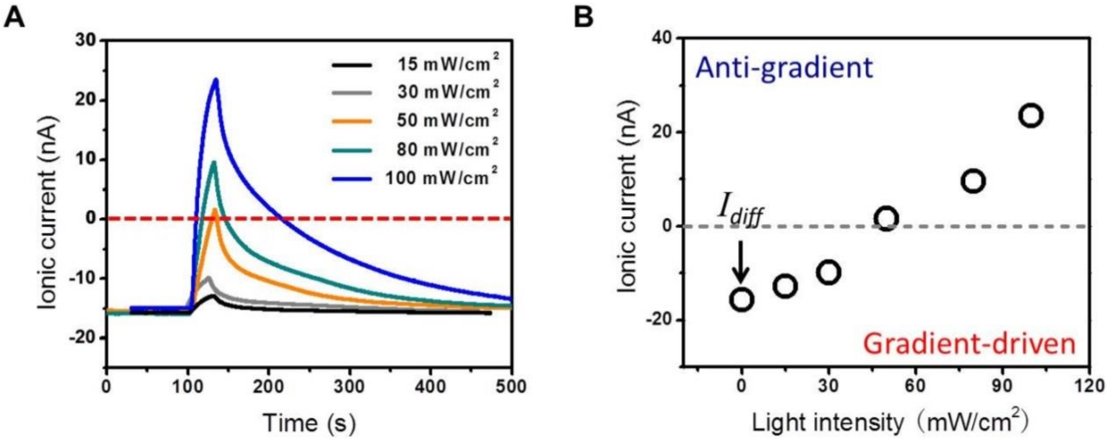

Supplementary Fig. 17. Influence of light intensity. **(A)** Time traces of the ionic current under asymmetric light illumination with varied intensities. **(B)** Peak value of the ionic current with respect to the light intensity. The illumination time was kept at 30 s. The concentration difference is C_{high}/C_{low}=10 μM/1 μM.

Supplementary Note 13. Experimental evidences for diffusion-controlled charge separation mechanism.

Firstly, we show the observed net ion transport upon asymmetric light illumination is driven by the light-induced electric potential difference (photovoltage). The photovoltage was measured in an open circuit state (Supplementary Fig. 18A). It keeps pace with the photocurrent. The polarity of the photovoltage depends on the illumination position (Supplementary Fig. 18B). The low electric potential is found in the illuminated side. The magnitude of the photovoltage goes up with the light intensity (Supplementary Fig. 18C).

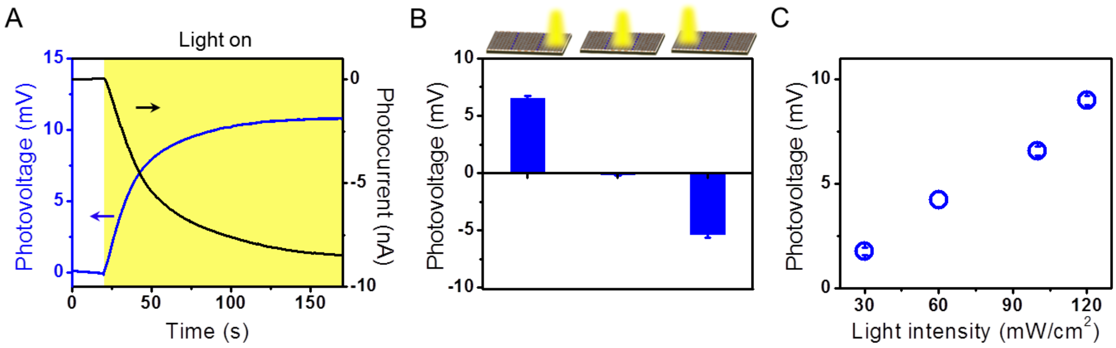

Supplementary Fig. 18. (A) Photovoltage and photocurrent versus illumination time. (B) Position dependence of the photovoltage. Illumination on three different positions (right, middle, or left) leads to different magnitudes and polarities of the photovoltage. The light intensity was 100 mW cm^{-2}, and the illumination time was 30 seconds. (C) Photovoltage measured under different light intensity. The electrolyte concentration was 1 μM. Error bars denote standard deviation.

Secondly, we show that the photovoltage is generated on the GOM (Supplementary Fig. 19). Metal evaporation was used for the fabrication of 80-nm-thick gold electrodes on the two ends of the dry GOM. Upon asymmetric light illumination, synchronized photovoltage is generated with peak value of about 10 mV in 30 seconds. The polarity of the photovoltage also depends on the illumination position. These features are in accord with that observed in wet GOM in ionic solution

(Supplementary Fig. 18). In addition, the linear dependence of the membrane resistance with respect to the membrane length reveals an ohmic-like contact at the electrode-GOM interface (Supplementary Fig. 20) [13]. This evidence excludes the possibility that the position-dependent photovoltage results from the Schottky barrier modulation at the electrode-GOM interface [14].

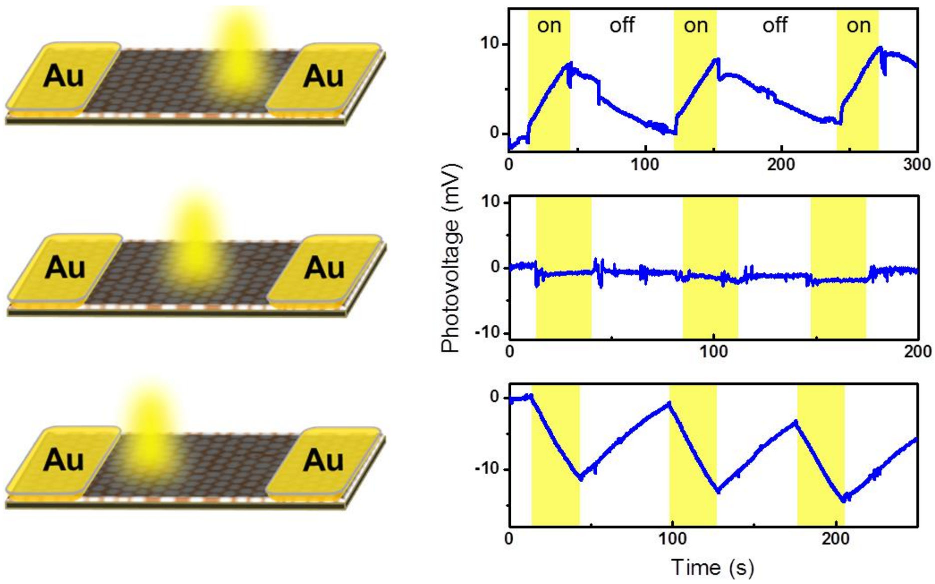

Supplementary Fig. 19. Photo-induced electric potential on dry GOM upon asymmetric illumination. Gold electrodes were fabricated by metal deposition at the two ends of the GOM. In all cases, the light intensity was 100 mW cm^{-2}.

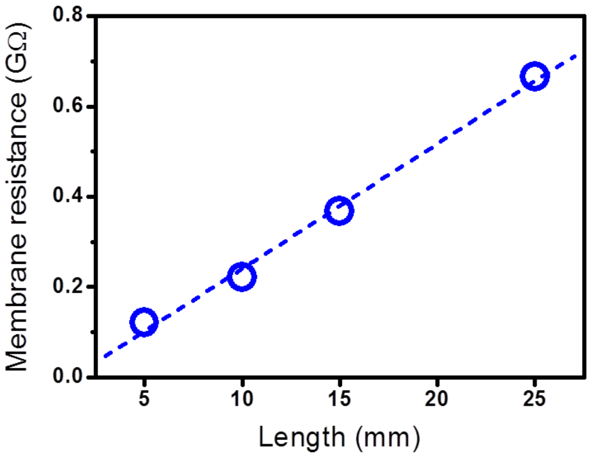

Supplementary Fig. 20. The membrane resistance increases linearly with the length of GOM, indicating an ohmic-like contact.

Thirdly, to explore the generation mechanism of the light-induced electrical potential difference along the GOM, we compare the conductance of dry GOM before and after full-area light illumination. The scheme of the testing device is shown in Supplementary Fig. 21A. The results show that the light illumination notably promotes the conductance of the GOM (Supplementary Fig. 21B and Supplementary Fig. 21C). The light illumination generates electron-hole pairs as extra charge carriers leading to the enhanced membrane conductance [15]. Increasing the illumination power further improves the carrier density, and thus promotes the conductance of GOM.

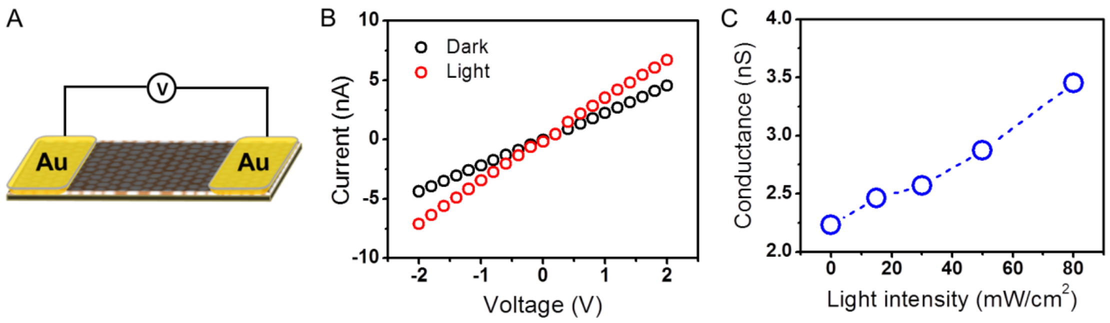

Supplementary Fig. 21. Photo-excited charge carriers in dry GOM. (A) Two gold electrodes were in contact with the GOM. Light illumination was applied to the full area of the GOM. (B) *I*–*V* characteristics of the dry GOM in dark and under illumination (80 mW cm^{-2}). (C) The membrane conductance increases with light intensity.

Fourthly, the photo-excited electron-hole pairs in GOM can be visualized by fluorescence imaging. Fluorescence emission spectrum (Princeton Instruments, SP2560) was used to characterize the photoluminescence of dry GOM. The dry GOM exhibits a broad fluorescence peak centered at 560 nm for laser excitation at 405 nm (Supplementary Fig. 22A), which is in agreement with previous report on the broad fluorescence emission band ranging from 400 to 800 nm [16]. The fluorescent signal results from the radiative recombination of photo-excited electron-hole pairs [17]. Further fluorescence imaging on the GOM was carried out with an inverted

microscope in confocal mode (Nikon, N-C2-SIM). The fluorescent signal was collected in the range of 510 to 560 nm with or without laser excitation at 488 nm. As shown in Supplementary Fig. 22B and Supplementary Fig. 22C, fluorescence from the GOM was observed upon light illumination, in sharp contrast to that observed in dark. The fluorescence intensity shows excellent linear relationship with the illumination power (Supplementary Fig. 22D).

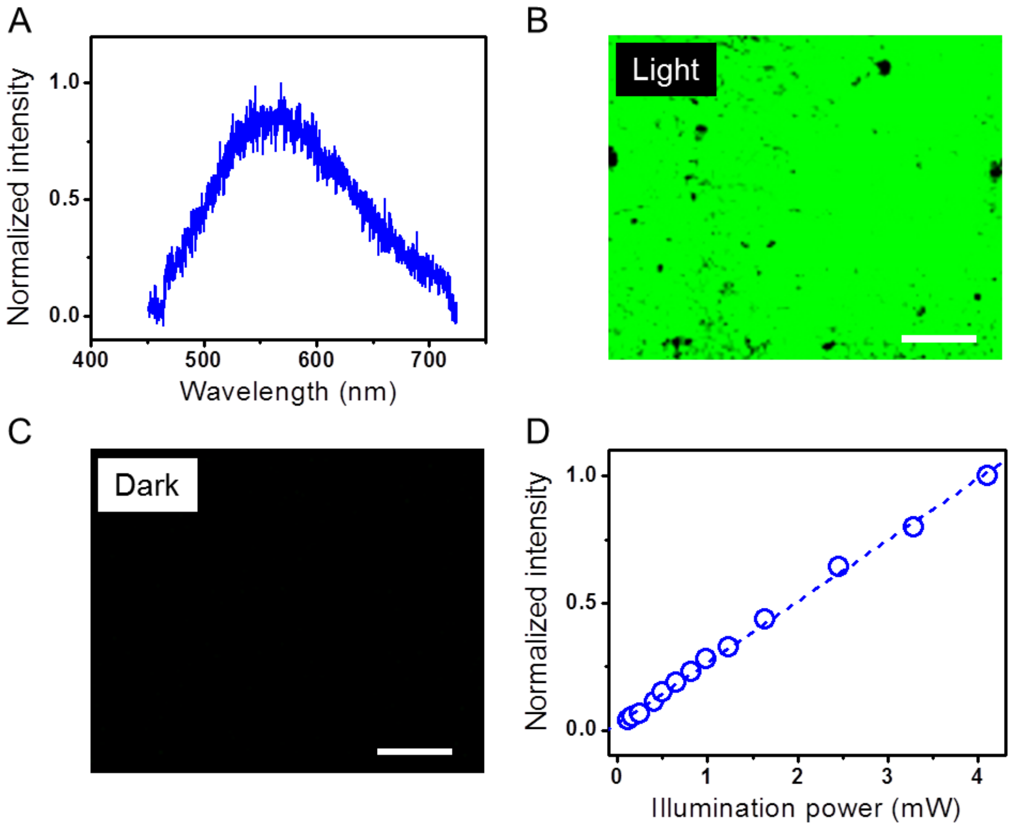

Supplementary Fig. 22. Fluorescence properties of dry GOM. (A) Fluorescence emission spectra of GOM excited at 405 nm. (B and C) False-colored fluorescence images of GOM with or without laser excitation at 488 nm. The scale bar was 50 μm. (D) The fluorescence intensity increases linearly with the illumination power.

We further show that the photo-induced potential difference on GOM is caused by asymmetric carrier diffusion. As shown in Supplementary Fig. 23, light illumination on the left 1/3 of a piece of dry GO strip generates a horizontal current. The direction of the photocurrent is from the illuminated region to the non-illuminated region. From previous results we know that light irradiation generates extra charge carriers (electrons and holes) in the illuminated region. These charge carriers would diffuse from the illuminated region to the non-illuminated region driven by their concentration gradients. In addition, for GO, the mobility of holes is higher than that of the electrons. Therefore, the asymmetric carrier diffusion results in a net current and potential difference.

Moreover, we add a small external voltage (V_{ext}) between the two ends of the GO strip (Supplementary Figs. 23A and 23B). When $V_{ext}>0$, the external electric field is in the same direction with the carrier diffusion. In this situation, the magnitude of the measured current is enhanced by the external electric field. This is because the parallel electric field accelerates the holes and slows down the electrons, which enhances the degree of charge separation. In contrast, when $V_{ext}<0$, the external electric field goes in the opposite direction with the carrier diffusion. In this situation, the magnitude of the photocurrent is reduced by the external electric field. This is because the anti-parallel electric field slows down the holes and accelerates the electrons, which undermines the charge separation. For comparison, we also test the influence of the electric field under full-area light illumination and in dark. As shown in Supplementary Figs. 23C and 23D, when $V_{ext}=0$, no measureable current was detected in these two conditions. The magnitude of current simply goes up with the electric field at both polarities.

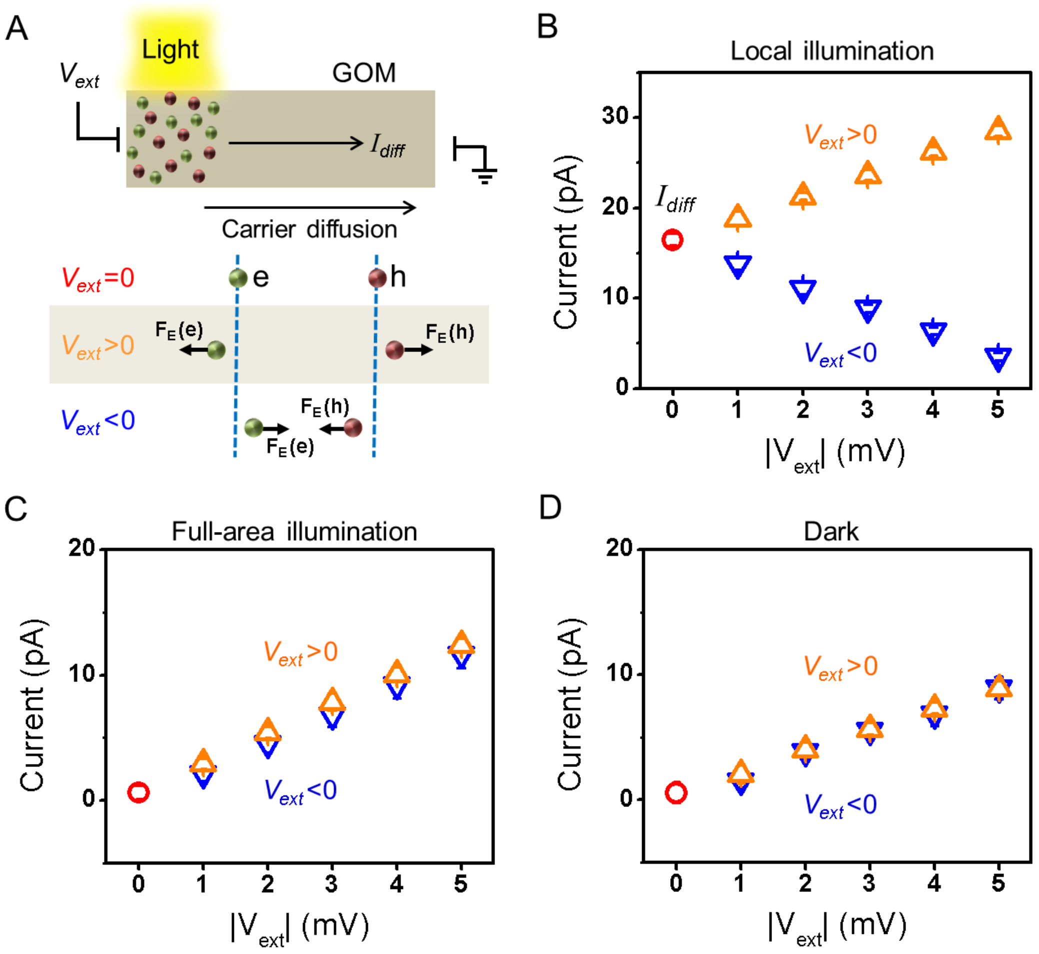

Supplementary Fig. 23. Direct measurement of current due to photo-generated carrier diffusion on dry GOM. A, scheme of the generation of carrier diffusion current upon local light illumination (50 mW cm^{-2}), and the influence of an externally applied electric field (V_{ext}) to the degree of charge separation. F_E is the force generated by V_{ext}. B-D, horizontal current measured upon local light illumination (B), full-area light illumination (C), and in dark (D). In C and D, the y-axis shows the absolute value of the current. Error bars denote standard deviation.

In general, light illumination on the GOM generates electron-hole pairs as extra charge carriers. When the light illumination was conducted locally on part of the GOM, the carrier density in the illuminated area would be higher than the non-illuminated area. Therefore, the excess charge carriers have the tendency to diffuse from the illuminated area to the non-illuminated area driven by its density

gradient. In this process, the intrinsic mobility difference between electrons and holes enables a diffusion-controlled charge separation [18], resulting in an unbalanced electric potential distribution between the illuminated area and the non-illuminated area. The unbalanced electric potential is the driving force for the photo-induced ion transport. We further develop a numerical model to quantify the diffusion-induced electric potential difference (see Supplementary Note 14 below).

Supplementary Note 14. Theoretical description of light-induced electric potential redistribution along GOM.

Light-induced change in local carrier concentration was described by an one dimensional continuity model (Supplementary Equation 5) that comprises the photo-excited carrier generation, recombination, diffusion, and electric-field-induced migration [19,20],

$$\frac{\partial \rho_e(x,t)}{\partial t} = gW(x,t) - h\rho_e(x,t)\rho_h(x,t) + D_e\frac{\partial^2 \rho_e(x,t)}{\partial x^2} - \mu_e\frac{\partial[\rho_e(x,t)\nabla\psi(x,t)]}{\partial x}$$
$$\frac{\partial \rho_h(x,t)}{\partial t} = gW(x,t) - h\rho_e(x,t)\rho_h(x,t) + D_h\frac{\partial^2 \rho_h(x,t)}{\partial x^2} + \mu_h\frac{\partial[\rho_h(x,t)\nabla\psi(x,t)]}{\partial x}$$
(5)

where the subscripts e and h denote electron and hole, respectively. ρ is the local carrier density, ψ is the local electric field, g is the photoresponsivity, W is the incident light intensity, h is the recombinant coefficient, D is the diffusion coefficient, and μ is the electric mobility. Boundary conditions were applied so that the net flow of each carrier is zero,

$$D_e\frac{\partial \rho_e(x,t)}{\partial x} - \mu_e\rho_e(x,t)\nabla\psi(x,t) = 0 \quad (6)$$

$$D_h\frac{\partial \rho_h(x,t)}{\partial x} + \mu_h\rho_h(x,t)\nabla\psi(x,t) = 0 \quad (7)$$

Supplementary Equation 5 was numerically integrated with a time step of 0.1 ps and initial densities of 1.0×10^{-9} mol m^{-1}. At each step, the electric field was obtained from the net charge distribution based on Gauss's law. The length of the model GO plate was set as 15 mm according to the experiment. The incident light spot was 5-mm-wide and centered at positions of x = -33.3%, -16.7%, 0%, +16.7%, and +33.3% (in length), respectively. The electric potential at infinity was set to 0 V. All parameters used in the calculation were collected from literatures listed in Supplementary Table 7. The diffusion coefficients were obtained based on Einstein relation, $\mu=2eD/k_BT_e$, where e and k_B are carrier charge and Boltzmann constants, T_e is carrier temperature [21].

Supplementary Table 7. Model parameters.

Parameters	Values	References
Photoresponsivity (g)	45 mA W^{-1}	[22]
Recombinant coefficient (h)	2.6×10^{12} m^2 $mol^{-1}s^{-1}$	[23,24]
Hole mobility (μ_h)	3.4×10^4 cm^2 $V^{-1}s^{-1}$	[21]
Electron mobility (μ_e)	6.8×10^3 cm^2 $V^{-1}s^{-1}$	[23,25]
Diffusion coefficient of hole (D_h)	5.5×10^3 cm^2 s^{-1}	$\mu=2eD/k_BT_e$
Diffusion coefficient of electron (D_e)	1.1×10^3 cm^2 s^{-1}	$\mu=2eD/k_BT_e$

The discrepant diffusion of e and h in GOM leads to an unbalanced net charge distribution (Supplementary Fig. 24). The net charge distribution depends on the illumination position. Upon asymmetric light illumination, a remarkable electric potential difference (ΔV) is built between the two ends of the model membrane (Supplementary Fig. 25). The calculated ΔV increases with the light intensity. When the light spot is more close to the edge of the GOM, the higher ΔV can be obtained.

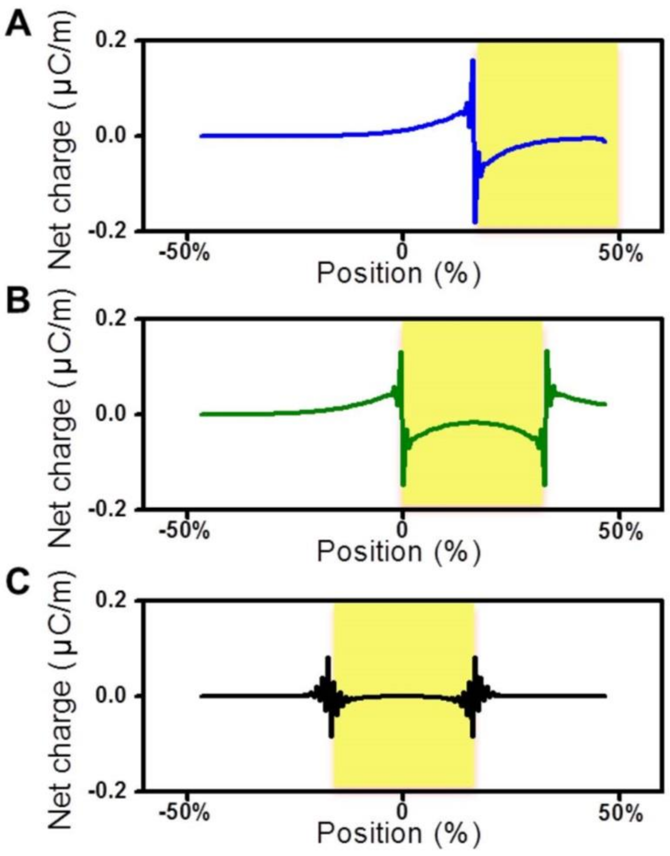

Supplementary Fig. 24. Net charge distribution along the model GOM upon local light illumination. The yellow shadow indicates the illuminated area. The light beam was centered at +33.3% (A), +16.7% (B), and 0% (C), respectively.

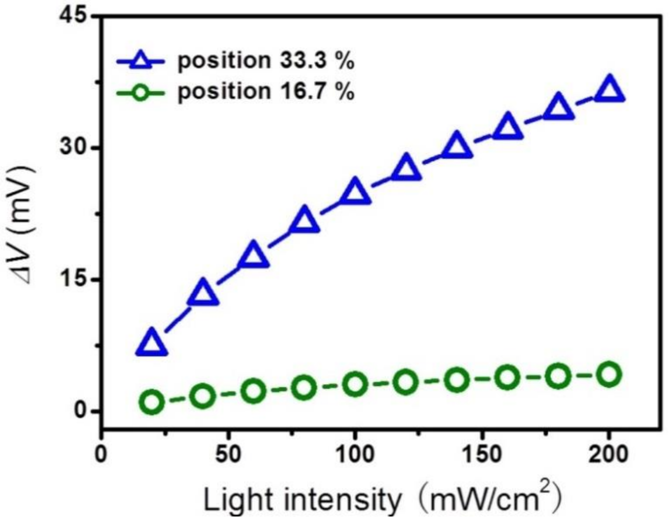

Supplementary Fig. 25. Photo-induced electric potential difference (Δ*V*) across the model GOM. The light beam was centered at +33.3% (triangle) and +16.7% (circle).

Supplementary Note 15. The role of carrier recombination.

In our model, the recombinant coefficient (*h*) describes the recombination rate of the charge carriers. With higher *h*, there would be less free charge carriers in each volume element, but the net charge density (numeric difference between electron and hole) therein gets larger (Supplementary Fig. 26A), reflecting a higher degree of charge separation. As a result, ΔV grows up with the increasing *h* (Supplementary Fig. 26B). From the net charge distribution shown in Supplementary Fig. 26A, one can see that the charge separation takes place mainly in the illuminated area, while outside the illuminated area, the net charge density quickly decays.

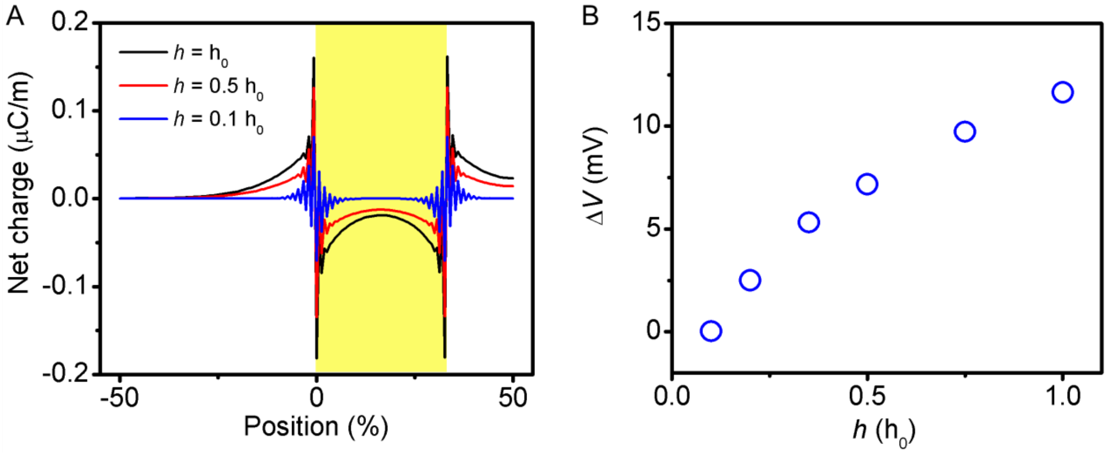

Supplementary Fig. 26. A, net charge distribution along the model GOM upon local light illumination. B, the calculated ΔV gradually grows up with the recombinant coefficient (*h*). The yellow shadow indicates the illuminated area (centered at +16.7%, width of 33%). h_0 refers to the recombinant coefficient used in this work (Supplementary Table 7). Other model parameters were identical with that used in Supplementary Note 14.

Supplementary Note 16. MD simulations of photo-induced active ion transport through GO nanochannel.

We performed MD simulations to investigate the ion transport behaviors inside the GO nanochannel under light illumination. The simulation box was shown in Supplementary Fig. 27. Two reservoirs (width = 3.2 nm, length = 4.4 nm, and height = 5.5 nm) were connected by a lamellar nanochannel (width = 3.2 nm, length = 10.0 nm, and height = 1.3 nm). The initial surface charge density on GO sheets was set to -25 mC m^{-2}, otherwise specifically mentioned. An imaged reservoir was employed to introduce periodic conditions in all directions. A certain number of water molecules and ions were added into the simulation box to keep charge neutral. All MD simulations were conducted in NAMD simulation package [26].

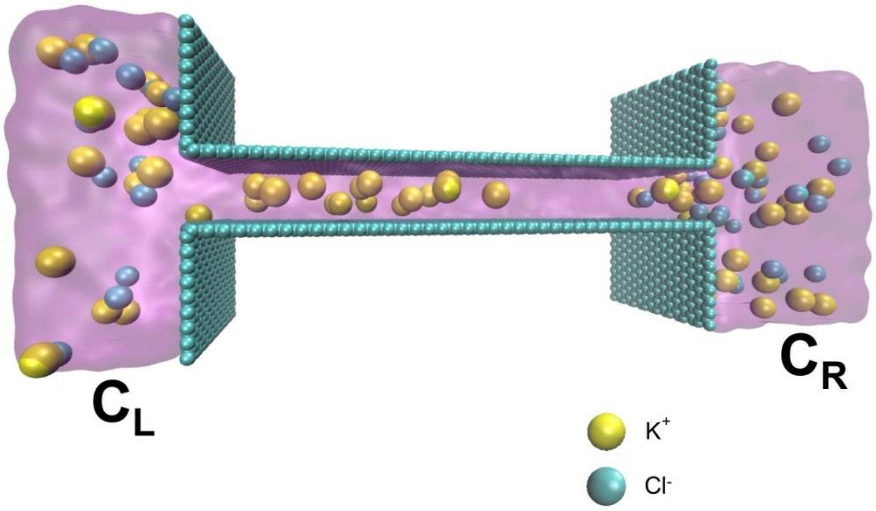

Supplementary Fig. 27. Schematic of the MD simulation structure drawn by VMD [27]. K^+ and Cl^- ions are in yellow and blue.

More simulation details can be found in previous works by us [28-30] and other groups [31,32]. Briefly, All-atoms charmm22 force field was used to describe bonded and non-bonded interactions among atoms [33]. For water molecules, SPC/E model was used. The cut-off distance for the L-J interaction was 1.2 nm. Long-range electrostatic interactions were computed via the particle mesh Ewald (PME) method with a cut-off

of 1.2 nm. Carbon atoms were frozen to their initial position. The SETTLE algorithm was used to maintain the geometry of the water molecules. The equilibrium density of the bulk water was around 1.0 g cm^{-3}. A leapfrog algorithm with a time step of 1.0 fs was used for time integration. Calculated trajectories were collected every 0.5 ps. The initial NPT simulations were conducted to equilibrate system for 2 ns at 300 K and 1 bar. Pressure was maintained by applying a Nosé-Hoover Langevin piston. And then NVT simulations were carried out for at least 10 ns for data collection at 300 K by applying a dual Langevin thermostat with a Langevin damping coefficient of 5 ps.

To simulate the influence of light illumination, we applied the photo-induced charge distribution onto the GO lamina for MD simulations. The net charge distribution under light illumination was calculated by the theoretical model mentioned above (Supplementary Note 14). The electrostatic potential was calculated by PMEpot plugins [34] in VMD by smooth particle-mesh Ewald method [35], which agrees well with the theoretical predictions. The resulting net ionic current was obtained by averaging the net ionic flux from 20 uniformly-distributed sections along the axial direction of the nanochannel.

Upon asymmetric illumination (100 mW cm^{-2}), the net flux of K^+ approaches ~2.2 $\times 10^8$ ions s^{-1} (Supplementary Fig. 28). No net ionic flux is found for symmetric illumination. The simulated photocurrent is perfectly contributed by cations ($t_+ \approx 1$, Supplementary Fig. 29A). In addition, the photo-induced ionic current increases with the surface charge density on GO sheets (Supplementary Fig. 29B).

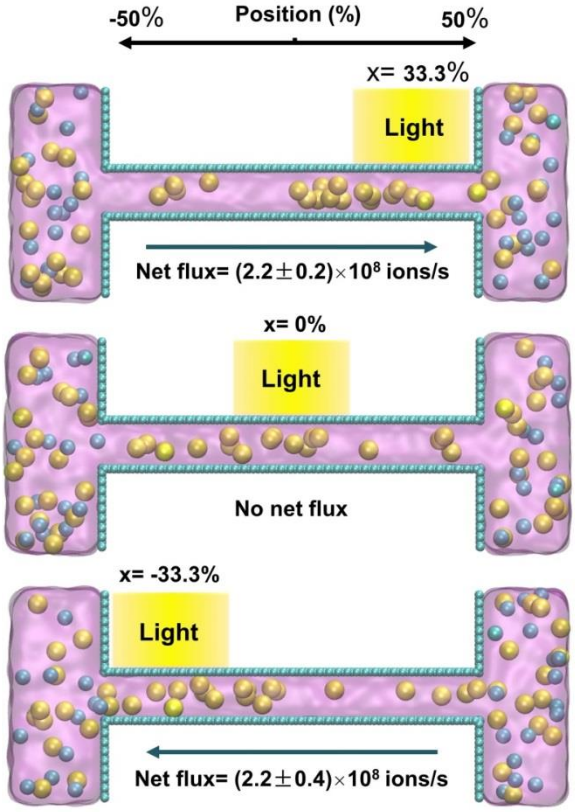

Supplementary Fig. 28. Light-induced ion transport within lamellar nanochannels. Potassium and chloride ions are denoted as yellow and blue balls, respectively. Due to the charge selectivity, chloride ions are expelled from the channel. Under asymmetric illumination (x = + 33.3% and −33.3%), potassium ions flow through the nanochannel from the non-illuminated area to the illuminated area.

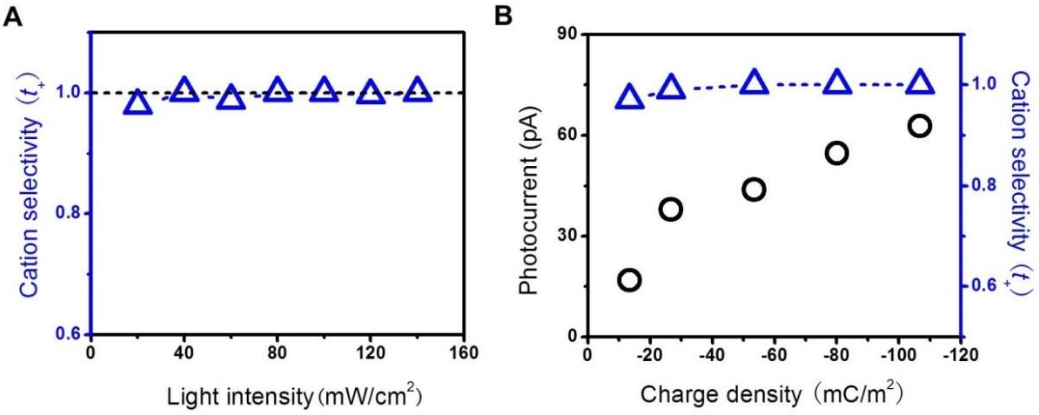

Supplementary Fig. 29. The photocurrent is unipolar (A) and surface-charge dependent (B).

Supplementary Note 17. Parameters for model calculation and MD simulations.

Supplementary Table 8. Model parameters for calculating photo-induced electric potential difference (ΔV).

Light intensity (mW cm^{-2})	Illumination position	ΔV (mV)	Data shown in the manuscript
100	-33.3%	-24.8	Fig. 3c
100	-16.7%	-3.3	Fig. 3c
100	0%	0	Fig. 3c
100	16.7%	3.3	Fig. 3c
100	33.3%	24.8	Fig. 3c
20	33.3%	7.6	Fig. 3d
40	33.3%	13.3	Fig. 3d
60	33.3%	17.6	Fig. 3d
80	33.3%	21.5	Fig. 3d
100	33.3%	24.8	Fig. 3d
120	33.3%	27.5	Fig. 3d
140	33.3%	30.0	Fig. 3d

Supplementary Table 9. Model parameters in MD simulation to calculate ion transport properties under light illumination.

Light intensity (mW cm^{-2})	Illumination position	C_H (M)	C_L (M)	Concentration difference (fold)	Photocurrent/ Ionic current (pA)	Data shown in the manuscript
100	-33.3%	1.0	1.0	1	-34.8	Fig. 3c
100	-16.7%	1.0	1.0	1	-2.4	Fig. 3c
100	0%	1.0	1.0	1	0.4	Fig. 3c
100	16.7%	1.0	1.0	1	2.2	Fig. 3c
100	33.3%	1.0	1.0	1	34.8	Fig. 3c
20	-33.3%	1.0	1.0	1	12.8	Fig. 3d
40	-33.3%	1.0	1.0	1	17.6	Fig. 3d
60	-33.3%	1.0	1.0	1	25.2	Fig. 3d
80	-33.3%	1.0	1.0	1	33.6	Fig. 3d
100	-33.3%	1.0	1.0	1	34.8	Fig. 3d
120	-33.3%	1.0	1.0	1	36.0	Fig. 3d
140	-33.3%	1.0	1.0	1	40.5	Fig. 3d
0	-33.3%	2.0	0.2	10	-10.41	Fig. 3e
20	-33.3%	2.0	0.2	10	-4.01	Fig. 3e
40	-33.3%	2.0	0.2	10	8.01	Fig. 3e
80	-33.3%	2.0	0.2	10	14.82	Fig. 3e
100	-33.3%	2.0	0.2	10	15.62	Fig. 3e
140	-33.3%	2.0	0.2	10	18.43	Fig. 3e
0	-33.3%	1.0	0.2	5	4.41	Fig. 3f

20	-33.3%	1.0	0.2	5	14.42	Fig. 3f
40	-33.3%	1.0	0.2	5	25.23	Fig. 3f
80	-33.3%	1.0	0.2	5	32.44	Fig. 3f
100	-33.3%	1.0	0.2	5	34.45	Fig. 3f
140	-33.3%	1.0	0.2	5	37.65	Fig. 3f
0	-33.3%	2.0	0.2	10	10.41	Fig. 3f
20	-33.3%	2.0	0.2	10	6.41	Fig. 3f
40	-33.3%	2.0	0.2	10	18.43	Fig. 3f
80	-33.3%	2.0	0.2	10	25.23	Fig. 3f
100	-33.3%	2.0	0.2	10	26.04	Fig. 3f
140	-33.3%	2.0	0.2	10	28.84	Fig. 3f
0	-33.3%	3.0	0.2	15	18.43	Fig. 3f
20	-33.3%	3.0	0.2	15	12.02	Fig. 3f
40	-33.3%	3.0	0.2	15	15.22	Fig. 3f
80	-33.3%	3.0	0.2	15	20.83	Fig. 3f
100	-33.3%	3.0	0.2	15	24.83	Fig. 3f
140	-33.3%	3.0	0.2	15	26.44	Fig. 3f

Supplementary Note 18. Experimental justification of theoretical predictions.

In the theoretical part, we propose that light illumination on GOM generates a localized low potential area. When the illuminated position is not in the center of the GOM, a potential difference is formed between the two ends of the membrane, which accounts for the observed active ion transport. In this part, we use an experimental method to justify the theoretical predictions.

Scheme of our device is shown in Supplementary Fig. 30. We placed three silicon electrodes with 300-nm-thick SiO_2 as dielectric layer atop the GOM, labeled as L (left), M (middle), and R (right). A negative gate voltage (V_G) of -3 V was applied separately on one of the electrodes, and the remaining two electrodes were used as reference potential (GND). In this way, we use a horizontally distributed electric potential to simulate the localized low potential area generated by light illumination. Other parts of the experimental setup were identical with that mentioned in the main text. Three configurations were investigated as shown in Supplementary Fig. 31A.

Before applying the gate voltage, no electric potential difference was applied across the GOM, and the recorded ionic current remains zero (Supplementary Fig. 31A). Right after applying V_G on the right electrode, a modulated ionic current is found synchronously. The direction of the modulated ionic current is from left to right. When V_G was applied on the left electrode, the modulated ionic current can be reversed. But, if V_G was applied on the middle electrode, no evident modulated ionic current can be detected. This trend is also summarized in Supplementary Fig. 31B. Moreover, the magnitude of the modulated ionic current goes up with the gate voltage (Supplementary Fig. 31C). These experimental evidences can be analogy to the photo-induced ionic current mentioned in the main text and justify our theoretical model.

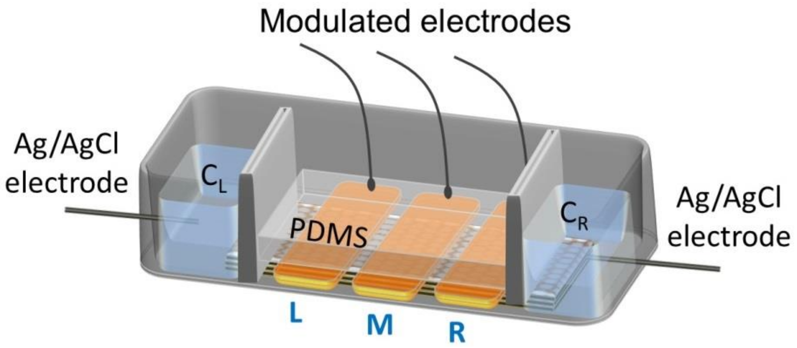

Supplementary Fig. 30. Setup for field effect modulation. Analogous to the photo-response experiment, we replace the light by a horizontally distributed electric potential.

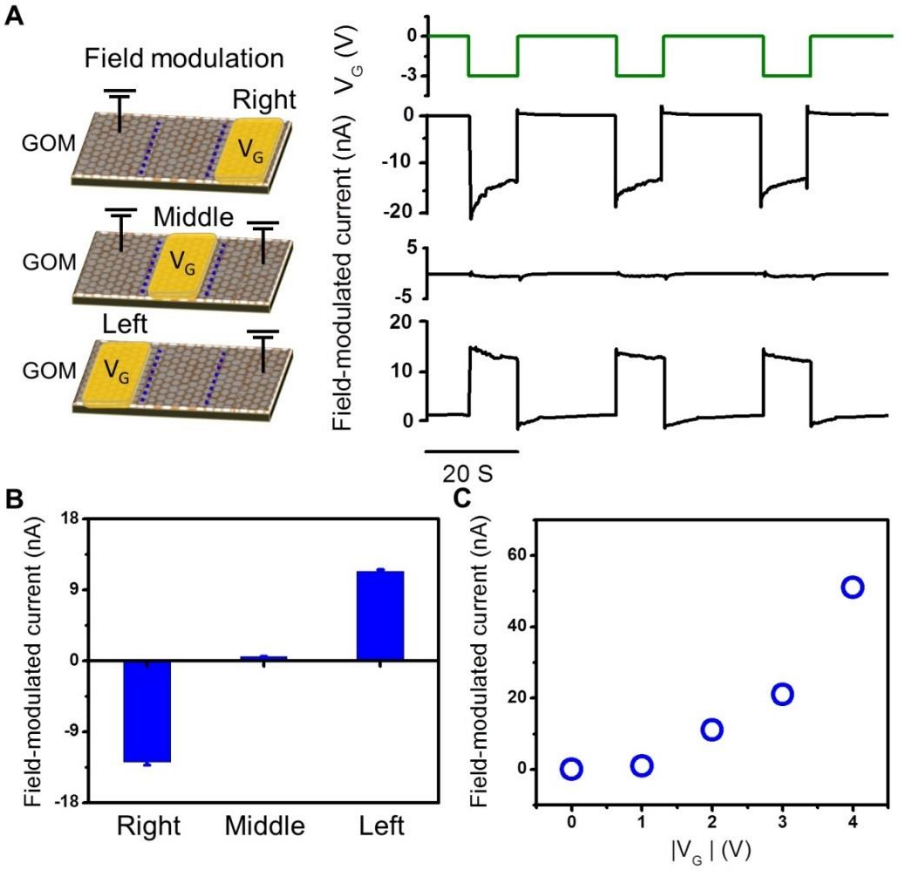

Supplementary Fig. 31. Field effect modulation of ionic current. **(A)** Time traces of the modulated ionic current with the applied gate voltage on different positions. **(B)** Summary of the position-dependent ionic current. Error bars denote standard deviation. **(C)** The modulated ionic current increases with the magnitude of gate voltage. The electrolyte solution was 1 μM KCl.

Supplementary Note 19. Consider carrier hopping in the model.

To achieve long-ranged ion transport through the entire membrane, carrier hopping across sheet boundaries should be considered in the model. As mentioned in previous reports [24,25], the inter-sheet carrier hopping might occur. We compare two models (Supplementary Fig. 32), the single-sheet model (without carrier hopping) and the multi-sheet model (with carrier hopping). In the multi-sheet model, GO nanosheets (500-nm-long) were uniformly arranged with 2 nm inter-sheet gap (Supplementary Fig. 32A). The total length of the GO channel was 15 mm for both the single-sheet model and the multi-sheet model. The light illumination was conducted on the left 1/3 of the two model channels. The photo-generated charge carriers were allowed to transport in the GO sheets and hop between adjacent sheets. The in-plane and inter-plane diffusion rates were separately considered as D_1 and D_2. This treatment is similar with that commonly used in organic photovoltaic devices, in which the intramolecular and intermolecular transport rates are scaled by separate diffusivities [36]. Experimentally, the value of D_1 is available in many literatures, but the value of D_2 for GO is not found in literatures. Therefore, we assume a simple linear relation between D_1 and D_2, $D_2 = \lambda D_1$ [37], where λ is a dimensionless coefficient ranging from 1 (the inter-plane transport is equally fast as the in-plane transport) to 0.001 (the inter-plane transport is three orders of magnitude slower than the in-plane transport). Then, the continuity equation was numerically integrated with a time step of 0.01 ps and initial charge carrier densities of 1.0×10^{-9} mol m^{-1}. At each step, the net charge distribution was used to calculate the electric field. The electric potential at infinity was set to zero as boundary conditions.

As shown in Supplementary Fig. 32B and Figure 3A, the multi-sheet model and the single-sheet model give similar photo-induced electric potential difference (ΔV) of about 25 mV between the two ends of the model channel, despite of the differences in the potential distribution. The polarity of ΔV depends on the illumination position. Under various light intensity, similar results are obtained (Supplementary Fig. 32C). In addition, the magnitude of ΔV shows weak negative correlation with the calculation

parameter λ (Supplementary Fig. 32D). With a decreasing λ from 1 to 0.001, the increment in ΔV is less than 1 mV. The multi-sheet model gives more precise description to the inter-sheet carrier transport and refines the electric potential profile. Both of the two models excellently support the experimental observations.

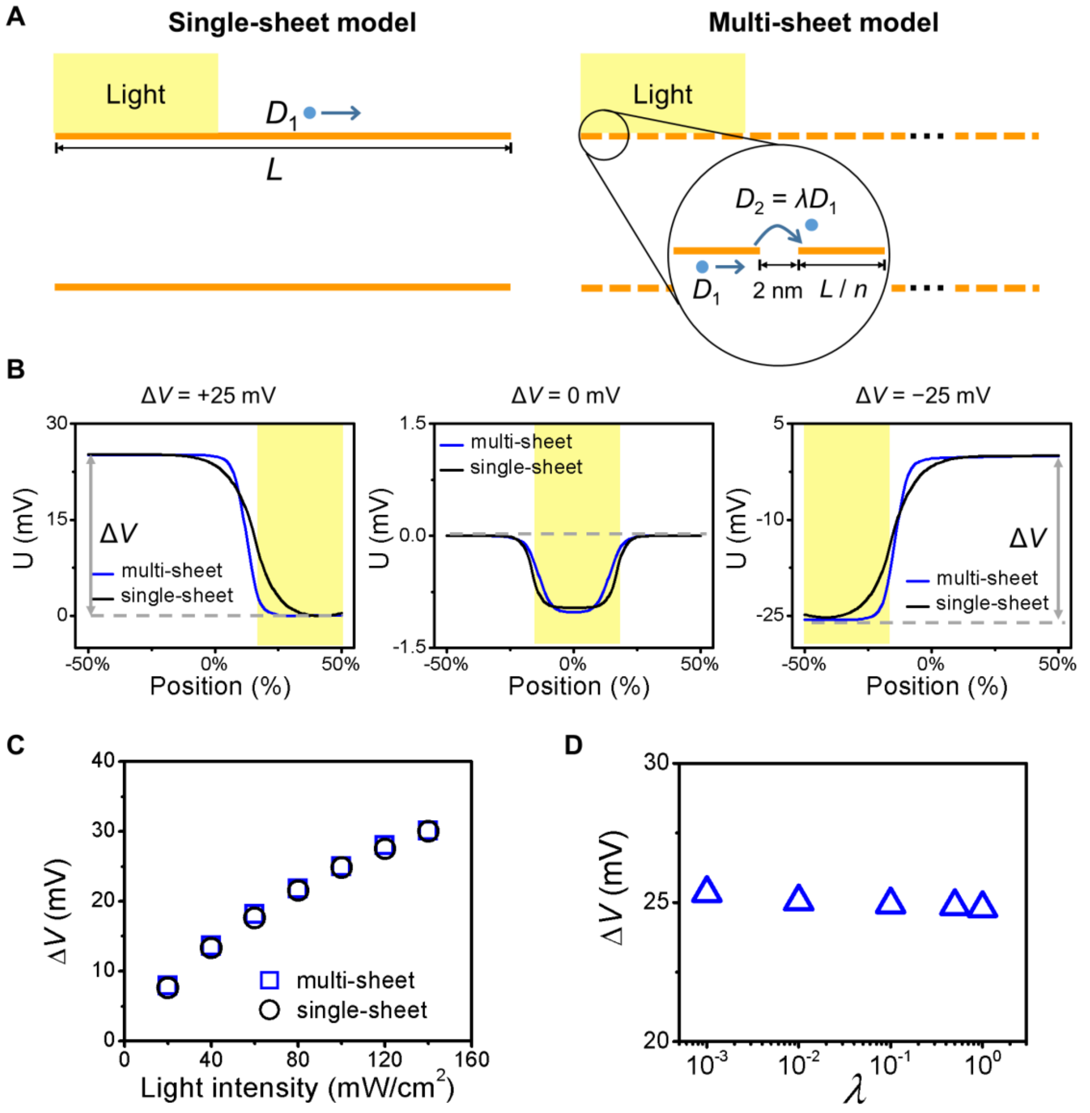

Supplementary Fig. 32. Influence of carrier hopping between GO nanosheets. (A) Scheme of the single-sheet and multi-sheet models. (B) Profiles of the electric potential predicted by the two models. The polarity of the potential difference between the two ends (ΔV) depends on the illumination position. (C) The two continuous models predict a similar dependence of ΔV as a function of the light intensity. (D) The calculated ΔV is insensitive to the value of λ.

Supplementary Note 20. Compare ion pumping rate with classical diffusion.

We compare the photo-induced ion pumping rate obtained from MD simulation with the estimated ion permeation rate from classical diffusion (Supplementary Fig. 33A). We applied a concentration difference across the model GO channel. The ionic concentration in the low-concentration (C_L) reservoir was 1 M, and the ionic concentration in the high-concentration (C_H) reservoir was $C_L+\Delta C$. In MD simulation, we applied a light beam on the right 1/3 of the GO channel, and calculate the ion transport rate of the cationic flow. The light intensity was 140 mW cm^{-2}. The simulation methods and other parameters were described in Supplementary Note 16. The size of the simulation box at lower ΔC might be varied to guarantee the accuracy. For comparison, we calculate the ion permeation rate via classical diffusion from the Fick's law at corresponding ΔC. The calculation method was described in Supplementary Note 8.

Under a series of concentration difference (ΔC) of 1, 0.5, 0.1, 0.03, 0.01, 0.003, and 0.001 M, respectively, the photo-induced ion transport is uphill against its concentration gradient, whereas, the concentration gradient driven ion permeation is downhill (Supplementary Fig. 33A). The two types of transport rate were summarized in Supplementary Table 11. The ion pumping rate largely exceeds the classical ion permeation rate under all tested ΔC. More intriguingly, we find that the ratio of the ion pumping rate to the estimated ion permeation rate exhibits an excellent linear relationship with respect to ΔC in the double logarithmic coordinates (R^2=0.9985, Supplementary Fig. 33B).

In this way, we can predict the ratio of transport rate in the experimental conditions by extrapolating the linear fitting curve to the low-concentration range. For example, in Figure 2d, ΔC was 9×10^{-6} M. The predicted ratio from the extended linear fitting curve was 6.8×10^{5}, i.e., the photo-induced ion pumping rate is five orders of magnitude higher than the classical ion permeation rate, which is in agreement with the experimental results shown in Supplementary Table 6 and Supplementary Fig. 13.

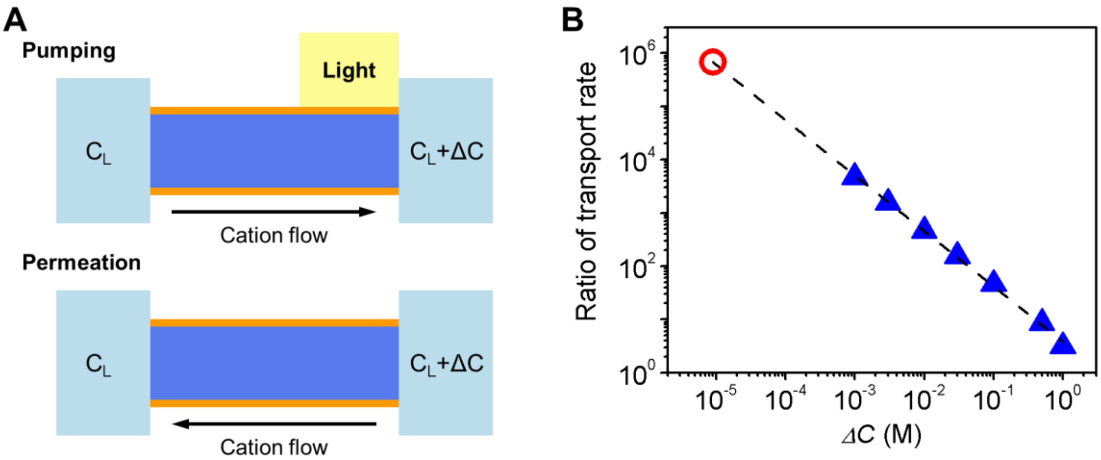

Supplementary Fig. 33. Comparison of transport rate between photo-induced ion pumping and classical ion permeation. (A) Schemes of the ion pumping (up) and permeation (down). A concentration difference (ΔC) was applied across the channel. The light intensity was 140 mW cm^{-2}. (B) The ratio of the ion pumping rate to the estimated ion permeation rate with respect to ΔC. They exhibit excellent linear relationship. The solid triangles were the calculated results, and the hollow circle was the predicted result from the extended linear fitting curve (dashed line).

Supplementary Table 10. Comparison of the transport rate between the photo-induced ion pumping and classical ion permeation.

ΔC (M)	Pumping rate (ions s^{-1})	Estimated permeation rate from classical diffusion (ions s^{-1})	Ratio of pumping rate and estimated permeation rate
1	1.58×10^8	5.01×10^7	3.15
0.5	2.15×10^8	2.50×10^7	8.60
0.1	2.30×10^8	5.01×10^6	45.98
0.03	2.32×10^8	1.50×10^6	154.65
0.01	2.34×10^8	5.01×10^5	467.19
0.003	2.35×10^8	1.50×10^5	1564.38
0.001	2.36×10^8	5.01×10^4	4701.88

Supplementary Note 21. Photo-modulation of ionic current.

Appropriate light illumination on the right part of the GOM blocks the ion transport driven by positive electric potentials (Supplementary Fig. 34A). While, appropriate light illumination on the left part blocks the ion transport driven by negative electric potentials (Supplementary Fig. 34B). The required light intensity increases with the applied voltage.

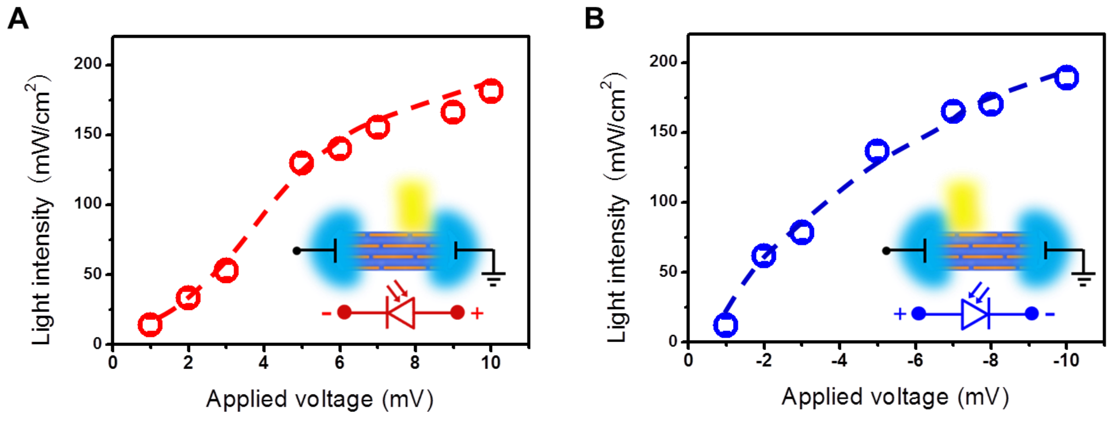

Supplementary Fig. 34. Light intensity required to switch-off the ionic current generated by positive **(A)** and negative voltage **(B)**. Error bars denote standard deviation.

The photoresponsivity (R) can be calculated from Fig. 4c as [15],

$$R = \frac{I_{ph}}{P_L} \tag{8}$$

where I_{ph} is the photocurrent ($I_{ph}= I_{illuminated} - I_{dark}$), and P_L is the incident light power. The photoresponsivity increases with light intensity. The highest photoresponsivity is ~6.4 μA W^{-1} (Supplementary Fig. 35).

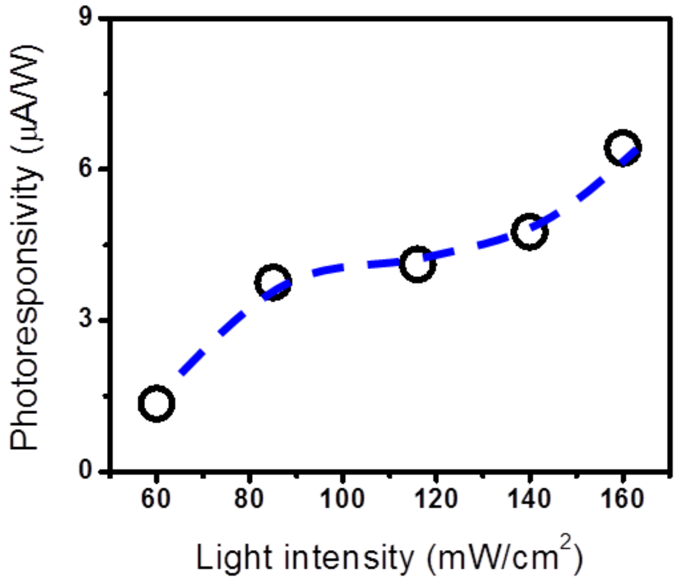

Supplementary Fig. 35. Photoresponsivity of the photonic ion transistor. The data was calculated from Fig. 4c at 1.0 V bias.

Supplementary Note 22. The equilibrium time.

We compare the equilibrium time between wet and dry GOMs. For the measurement on dry GOM, gold electrodes were evaporated on the two ends of the membrane. While for wet GOM, the measurement was conducted in the electrochemical cell. Typical voltage-time curves were shown in Supplementary Fig. 36A and 36B. We further quantify the response time of the voltage trace by fitting the experimental data with an exponential function [38],

$$V = V_0 + A \exp\left(\frac{-t}{\tau}\right) \tag{9}$$

where τ was the time constant, V_0 and A were fitting parameters. At a given light intensity, the time constants corresponding to wet and dry GOMs are close (Supplementary Fig. 36C), approaching tens of seconds. This evidence suggests that the relatively long equilibrium time may not result from the ion transport behaviors in solution.

In addition, we also test the light transmittance through the GOMs in both wet and dry states (Supplementary Fig. 36D). The GOMs were about 5.1 μm thick in dry state (used throughout this work). The light transmittance approaches 28.2%. This evidence suggests that the light intensity in the depth direction is not homogenous. The inhomogeneous light irradiation in the depth direction may influence the establishment of a steady-state electric potential distribution in GOM.

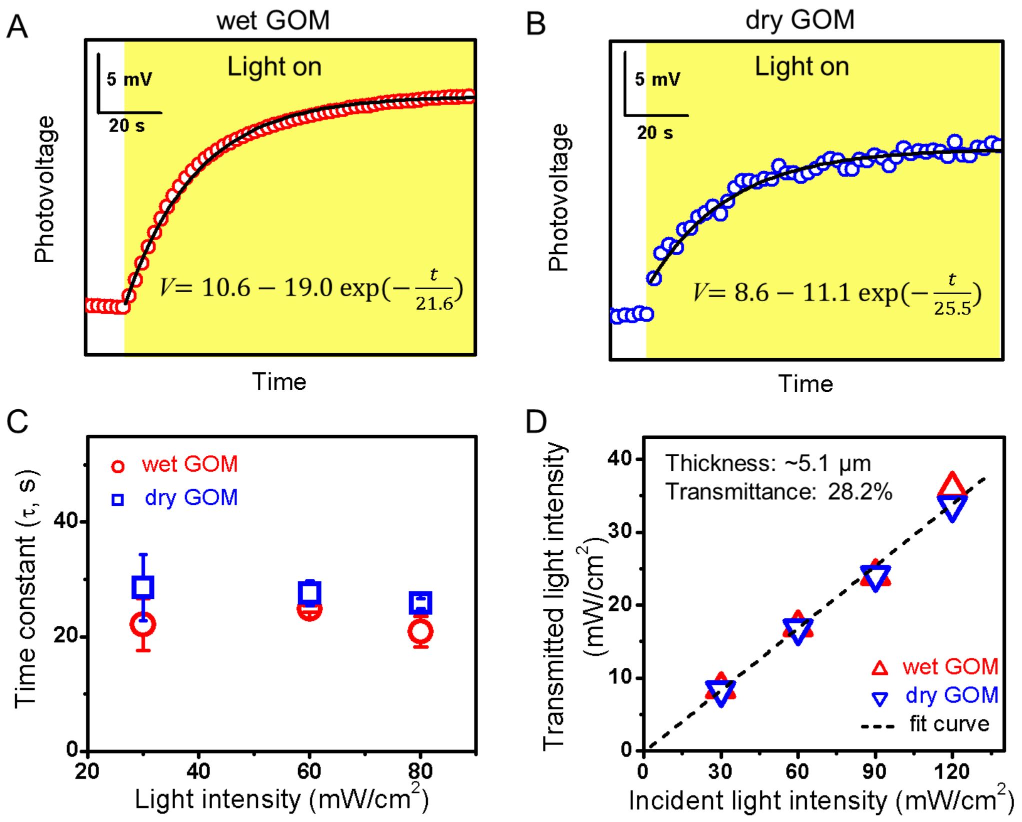

Supplementary Fig. 36. A and B, typical time traces of photovoltage on wet and dry GOMs. The voltage-time curves can be numerically fitted with an exponential function. C, the time constant (τ) obtained at different light intensity. Error bars denote standard deviation. D, transmitted light intensity through wet and dry GOMs. The membrane thickness was about 5.1 μm.

Supplementary References.

1 Joshi, R. *et al.* Precise and ultrafast molecular sieving through graphene oxide membranes. *Science* **343**, 752-754 (2014).

2 Gao, J. *et al.* High-performance ionic diode membrane for salinity gradient power generation. *J. Am. Chem. Soc.* **136**, 12265-12272 (2014).

3 Kate, O., Jong, M., Hintzen, H. & Kolk, E. Efficiency enhancement calculations of state-of-the-art solar cells by luminescent layers with spectral shifting, quantum cutting, and quantum tripling function. *J. Appl. Phys.* **114**, 084502 (2013).

4 Bennett, I. *et al.* Active transport of Ca^{2+} by an artificial photosynthetic membrane. *Nature* **420**, 398-401 (2002).

5 Xie, X., Crespo, G., Mistlberger, G. & Bakker, E. Photocurrent generation based on a light-driven proton pump in an artificial liquid membrane. *Nat. Chem.* **6**, 202-207 (2014).

6 Li, T. *et al.* Thermoelectric properties and performance of flexible reduced graphene oxide films up to 3,000 K. *Nat. Energy* **3**, 148-156 (2018).

7 Eda, G. & Chhowalla, M. Graphene-based composite thin films for electronics. *Nano Lett.* **9**, 814-818 (2009).

8 Chang, H. *et al.* Thin film field-effect phototransistors from bandgap-tunable, solution-processed, few-layer reduced graphene oxide films. *Adv. Mater.* **22**, 4872-4876 (2010).

9 dos Santos, P., Timm, R., Kubota, L. & Bonacin, J. Modulation of electrochemical properties of graphene oxide by photochemical reduction using UV-light emitting diodes. *ChemistrySelect* **1**, 1168-1175 (2016).

10 Pei, S. & Cheng, H.-M. The reduction of graphene oxide. *Carbon* **50**, 3210-3228 (2012).

11 Abraham, J. *et al.* Tunable sieving of ions using graphene oxide membranes. *Nat. Nanotechnol.* **12**, 546-550 (2017).

12 Li, D., Muller, M., Gilje, S., Kaner, R. & Wallace, G. Processable aqueous dispersions of graphene nanosheets. *Nat. Nanotechnol.* **3**, 101-105 (2008).

13 Kaiser, A., Gómez-Navarro, C., Sundaram, R., Burghard, M. & Kern, K. Electrical conduction mechanism in chemically derived graphene monolayers. *Nano Lett.* **9**, 1787-1792 (2009).

14 Ghosh, S., Sarker, B., Chunder, A., Zhai, L. & Khondaker, S. Position dependent photodetector from large area reduced graphene oxide thin films. *Appl. Phys. Lett.* **96**, 163109 (2010).

15 Lopez-Sanchez, O., Lembke, D., Kayci, M., Radenovic, A. & Kis, A. Ultrasensitive photodetectors based on monolayer MoS_2. *Nat. Nanotechnol.* **8**, 497-501 (2013).

16 Shang, J. *et al.* The origin of fluorescence from graphene oxide. *Sci. Rep.* **2**, 792 (2012).

17 Loh, K., Bao, Q., Eda, G. & Chhowalla, M. Graphene oxide as a chemically tunable platform for optical applications. *Nat. Chem.* **2**, 1015-1024 (2010).

18 Chen, R. *et al.* Charge separation via asymmetric illumination in photocatalytic Cu_2O particles. *Nat. Energy* **3**, 655-663 (2018).

19 Vicarelli, L. *et al.* Graphene field-effect transistors as room-temperature terahertz detectors. *Nat. Mater.* **11**, 865-871 (2012).

20 Dyakonov, M. & Shur, M. Shallow water analogy for a ballistic field effect transistor: new mechanism of plasma wave generation by dc current. *Phys. Rev. Lett.* **71**, 2465-2468 (1993).

21 Ruzicka, B. *et al.* Hot carrier diffusion in graphene. *Phys. Rev. B* **82**, 195414 (2010).

22 Qi, X. *et al.* Ultraviolet, visible, and near infrared photoresponse properties of solution processed graphene oxide. *Appl. Surf. Sci.* **266**, 332-336 (2013).

23 Li, S., Tu, K., Lin, C., Chen, C. & Chhowalla, M. Solution-processable graphene oxide as an efficient hole transport layer in polymer solar cells. *ACS Nano* **4**, 3169-3174 (2010).

24 Eda, G., Mattevi, C., Yamaguchi, H., Kim, H. & Chhowalla, M. Insulator to semimetal transition in graphene oxide. *J. Phys. Chem. C* **113**, 15768-15771 (2009).

25 Gómez-Navarro, C. *et al.* Electronic transport properties of individual chemically reduced graphene oxide sheets. *Nano Lett.* **7**, 3499-3503 (2007).

26 Phillips, J. C. *et al.* Scalable molecular dynamics with NAMD. *J. Comput. Chem.* **26**, 1781-1802 (2005).

27 Humphrey, W., Dalke, A. & Schulten, K. VMD: visual molecular dynamics. *J. Mole. Graph.* **14**, 33-38 (1996).

28 Chen, Q., Kong, X., Li, J., Lu, D. & Liu, Z. Electrokinetic desalination using honeycomb carbon nanotubes (HC-CNTs): a conceptual study by molecular simulation. *Phys. Chem. Chem. Phys.* **16**, 18941-18948 (2014).

29 Li, J., Kong, X., Lu, D. & Liu, Z. Italicized carbon nanotube facilitating water transport: a molecular dynamics simulation. *Sci. Bull.* **60**, 1580-1586 (2015).

30 Lu, D. N. Accelerating water transport through a charged SWCNT: a molecular dynamics simulation. *Phys. Chem. Chem. Phys.* **15**, 14447-14457 (2013).

31 Hummer, G., Rasaiah, J. & Noworyta, J. Water conduction through the hydrophobic channel of a carbon nanotube. *Nature* **414**, 188-190 (2001).

32 Algara-Siller, G. *et al.* Square ice in graphene nanocapillaries. *Nature* **519**, 443-445 (2015).

33 MacKerell Jr, A. *et al.* All-atom empirical potential for molecular modeling and dynamics studies of proteins. *J. Phys. Chem. B* **102**, 3586-3616 (1998).

34 Aksimentiev, A. & Schulten, K. Imaging alpha-hemolysin with molecular dynamics: ionic conductance, osmotic permeability, and the electrostatic potential map. *Biophys. J.* **88**, 3745-3761 (2005).

35 Essmann, U. *et al.* A smooth particle mesh ewald method. *J. Chem. Phys.* **103**, 8577-8593 (1995).

36 Liang, C. *et al.* Modeling and simulation of bulk heterojunction polymer solar cells. *Solar Energy Mater. Solar Cell.* **127**, 67-86 (2014).

37 Tsen, A. *et al.* Tailoring electrical transport across grain boundaries in polycrystalline graphene. *Science* **336**, 1143 (2012).

38 Chitara, B., Panchakarla, L., Krupanidhi, S. & Rao, C. Infrared photodetectors based on reduced graphene oxide and graphene nanoribbons. *Adv. Mater.* **23**, 5419-5424 (2011).
